# Factors impacting the implementation of a psychoeducation intervention within the mental health system: a multisite study using the consolidation framework for implementation research

**DOI:** 10.1186/s12913-020-05852-9

**Published:** 2020-11-09

**Authors:** Agnes Higgins, Rebecca Murphy, Carmel Downes, Jennifer Barry, Mark Monahan, David Hevey, Thilo Kroll, Louise Doyle, Patrick Gibbons

**Affiliations:** 1 School of Nursing & Midwifery, Trinity College Dublin, 24 D’Olier Street, Dublin, Ireland; 2 Department of Psychology, Maynooth University, Kiladre, Ireland; 3 School of Psychology, Trinity College Dublin, Dublin, Ireland; 4 School of Nursing, Midwifery and Health Systems, University College Dublin, Dublin, Ireland; 5 Celbridge Adult Mental Health Services, Kildare, Ireland

**Keywords:** Implementation science, Barriers, Enablers, Psychoeducation, Mental health services, Framework analysis, Consolidation framework for implementation research

## Abstract

**Background:**

Despite a strong evidence base and policy recommendation supporting the implementation of psychoeducation interventions within the mental health system, equitable access for many service users and family members has not been achieved. To enhance translation, developing an evidence-base around the factors that influence implementation of interventions is critical.

**Methods:**

The aim of the study was to explore the factors influencing implementation of a group cofacilitated recovery focused psychoeducation intervention. The study design was explorative qualitative descriptive, involving the collection of data through individual and focus group interviews with key stakeholders (*n* = 75) involved with the implementation within 14 mental health sites in the Republic of Ireland. The Consolidation Framework for Implementation Research (CFIR) was used as a conceptual framework to guide data collection and analysis.

**Results:**

Key enablers and barriers were identified across all CFIR domains of the framework with some factors (depending on context) being both an enabler and a barrier. Important factors in the outer setting domain included structural stability within national systems and the peer payment system, while the extent of a recovery-oriented culture, leadership, implementation readiness, and buy-in were influential factors in the inner setting. The characteristics of the intervention in terms of design, evidence-base and adaptability also shaped the intervention’s implementation as did the knowledge, beliefs and self-efficacy of facilitators. In terms of processes, implementation was influenced by the degree of engagement of key individuals who championed and supported the programme. The results highlight that while some of the barriers were specific to the programme, many reflected systemic and structural challenges within health services more generally.

**Conclusion:**

Findings from this study provide an enhanced understanding of the different layers of determinants to implementation of an intervention. Overcoming challenges will involve positive and ongoing engagement and collaboration across the full range of stakeholders that are active within each domain, including policy and operational levels. The quality of leadership at each domain level is of crucial importance to successful implementation.

**Supplementary Information:**

**Supplementary information** accompanies this paper at 10.1186/s12913-020-05852-9.

## Background

In response to the global mental health policy aspiration of ‘recovery-oriented care’ [1, 2], the epistemological orientation of Mental Health Systems (MHS) are undergoing a period of tremendous change. Meaningful realisation of the recovery ethos into routine practice mandates systemic shifts at structural, organisational and practice levels. Entrenched traditional models of care in MHS are being reorganized to re-orient the service ethos towards the promotion of hope and self-determination, and strengthen competencies in developing egalitarian partnerships and co-production [3]. In some countries significant investment into developing, implementing and evaluating pro-recovery interventions has ensued including peer support services, advanced directives, Wellness Recovery Action Plans (WRAP), illness management and recovery programs (IMR) and so on [4, 5]. A progressive portfolio of empirical research examining service and client outcomes, such as efficacy, efficiency, safety, and acceptability, have emerged to inform consensus appraisal of evidence-based practice and the development of intervention manuals and best practice guides. However, like most human systems, mental health services are resistant to change, and while this extensive empirical evidence-base is building consensus on best practice [6, 7], many countries continue to report that there “has been more progress made in envisioning a ‘recovery oriented system of care’ than in implementing one” [[8] p.1094].

Two decades ago, Grol and Grimshaw [9] made a request for evidence-based practice to be complemented by evidence-based implementation, a plea arising from the notoriously lengthy time it took for research to be translated into practice and/or policy. Indeed, a recent review estimated that it takes 17 years for just 14% of original research evidence to be instituted into clinical practice [10]. The research on health interventions has provided little evidence about why similar interventions succeed or fail in different settings. Consequently, lack of knowledge about key determinants that inhibit or enable implementation can result in wide variations in the manner in which an intervention is implemented.

Despite these shortcomings, the science of Dissemination and Implementation (D&I) is advancing, with increased funding directed towards narrowing the longstanding phenomenon sometimes referred to as the “efficacy-effectiveness gap” [11]. Today there is a greater appreciation that demonstrating effectiveness is insufficient to promote adoption and sustainability of evidence-based interventions [12]. To enhance the uptake and embedding of innovations into everyday practice, a broader research focus is required, one that extends beyond effectiveness to encompass the inter-related and multi-level contextual factors (e.g. related to the intervention, individual, implementer/provider, organizational, policy levels) which dynamically interact in the real-world, natural environment of the health system [13, 14].

Similar to all non-pharmacological interventions in mental health care, the translation of psychoeducation’s proven efficacy into routine mental health care has endured a protracted and challenging path. Psychoeducation as an evidence-based intervention aims to inform people about interventions and a self-management approach to the care and treatment of their or their family member’s mental health problem, with a view to enhancing communication, problem-solving and coping skills [15–18]. Despite the strength of the evidence-base in relation to psychoeducation [19–24], and its successful integration into policy recommendation and clinical guidelines [25–28], equitable access for many service users and family members has still not been achieved [29]. Alongside nascent research exploring the implementation determinants of one to one psychoeducation [30–33], a recent scoping review [34] reported some participant, practitioner, intervention, organisational and structural barriers and facilitators to group psychoeducation. While the review provided useful insights, the authors noted several methodological limitations of the included studies which restrict the generalisability of their findings, including heterogeneity of study design and a narrow focus on one stakeholder perspective and one site of implementation. In addition, they highlighted the absence of studies informed by an implementation theory or framework, which resulted in limited cohesion in study findings and unexplored fields of inquiry.

In order to build a stronger evidence-base and thus facilitate the development and testing of implementation strategies, there is a need for more robust, larger-scale studies, informed by theories of implementation. Indeed, given the assertion that implementation is the bridge between the decision to adopt an intervention and its routine use in practice [[35] p.3], developing an evidence-base around the factors that influence the implementation of group psychoeducation interventions is critical. To address this issue, with the support of grant funding, this study utilised the Consolidated Framework for Implementation Research (CFIR) [35] to examine determinants affecting the implementation of two group psychoeducation programmes for service users and family members, into mainstream mental health services in the Republic of Ireland.

### EOLAS intervention

The intervention (called EOLAS, which is the Irish word for knowledge) consists of two parallel structured psychoeducation information programmes (one for people who have been diagnosed with schizophrenia spectrum or bipolar disorders and a seperate programme for family members and other supporters). Both programmes were developed in collaboration with service users and family members and are jointly facilitated by peers (service users and family members) and clinicians [36], thus combining lived experience with clinical knowledge and expertise. The programmes consist of 8 weekly sessions of approximately 90-min duration, with some sessions being delivered by a guest speaker who has been selected in conjunction with the participants. To date, that is usually a psychiatrist but other clinicians, such as pharmacologists and psychologists, have also been guest speakers. Potential participants are referred to the programme by members of the multidisciplinary Community Mental Health Team. All facilitators (clinicians and peers) undergo a four-day training programme, where they learn about the programme and are supported to develop co-facilitation skills. In addition to facilitators having a handbook to guide them through the programme, participants also receive a handbook, which they can take home and use as a resource of information after completing the programme. Maintaining fidelity of the programmes to the EOLAS model is overseen by a Steering Committee that is representative of relevant stakeholders, including service users, family representatives, clinicians, academics and funders. Funding to pay national project workers, peer facilitators’ stipends, training of facilitators and publication of handbooks is provided by the Health Service Executive (HSE), the statutory agency tasked with the delivery and management of the public health services in Ireland. More detailed information on the co-design, content, facilitator training and impact of the EOLAS programmes has been reported previously [21, 36–39].

## Methodology

### Aim

The aim of this current aspect of the study was to explore the factors influencing implementation of the EOLAS programmes.

### Ethical approval

The University’s Research Ethics Committee (Reference Number 170901) and the ethics committees of the services involved provided ethical approval. The Director of Nursing at each site provided permission to recruit participants within their service following communication with a member of the research team.

## Methods

While a randomised control trial is considered a gold standard for evaluating the effectiveness of an intervention [40], it is limited in its usefulness in understanding factors that influence the implementation process for psychosocial interventions. Therefore, the study design for the current study was explorative qualitative descriptive, involving the collection of data through individual and focus group interviews. Qualitative descriptive research is directed at providing an in-depth description of an experience or event [41] and enables researchers to develop a deep understanding of the phenomena under study. The Consolidation Framework for Implementation Research (CFIR) [35] was used as a conceptual framework to guide data collection and analysis. The CFIR is a meta-theoretical framework designed to guide the appraisal of implementation contexts, including the factors that might influence implementation. The framework consists of five domains: outer setting, inner setting, intervention characteristics, individual (provider) characteristics and implementation process. Table 1 provides an overview of the framework.

**Table 1:** Overview of the Consolidation Framework for Implementation Research

Domain	Meaning
Outer setting	The wider social/political/economic context in which the organisation is embedded. The focus of this domain is on the organisation’s/service’s understanding of, and responsiveness to, patient needs, its links with other external organisations, and its competitiveness with peers who have already implemented the intervention as well as the influence of external policies, regulations and incentives designed to promote the intervention.
Inner setting	The structural and cultural characteristics of the organisation/service where the intervention is implemented. This domain addresses the constructs of: culture, leadership, mentoring, networking, communication patterns, the implementation climate in terms of whether practitioners collectively work towards implementing the intervention and the implementation readiness of the service in terms of leadership engagement and resource availability.
Intervention	Intervention refers to the characteristics of the intervention, and focuses on stakeholders’ views about the quality of the evidence about the intervention, the quality of the design, its ability to adapt to local needs, how it compares to other interventions and how difficult it is to implement. Also includes costs associated with the intervention and its ability to be piloted.
Characteristics of individuals (provider)	The constructs within this domain include: a person’s knowledge and beliefs about the intervention, their self-efficacy in relation to providing the intervention, their commitment to their organisation/service, and other personal attributes that may impact implementation, such as motivation, competence, nature of work contract, and past experiences.
Implementation process	The implementation domain addresses activities undertaken as part of the implementation process and includes the constructs of planning, engagement of appropriate individuals, the quality of the execution process and evaluation methods.

Damschroder LJ, Aron DC, Keith RE, Kirsh SR, Alexander JA, Lowery JC. Fostering implementation of health services research findings into practice: a consolidated framework for advancing implementation science. Implementation Science. 2009;4(1):50

### Data collection

Data were collected using semi-structured, audio recorded interviews (one to one and focus group). Due to limited resources and a desire to capture the views of as many participants as possible from services that were geographically spread, the research group decided to provide potential participants with the option of either a focus group interview or an individual interview. The option of a focus group also facilitated the collection of data from potential participants who were meeting as a group for other EOLAS related issues. The purpose of the interview was to explore participants’ views on the factors they believed enabled or hindered the implementation of the intervention. The team developed an interview schedule to guide data collection which was based on the Consolidation Framework for Implementation Research (CFIR) [35]. The interview guides developed for this study are provided as supplementary materials (Additional Files 1, 2, 3, 4, 5). To ensure consistency in data collection the same schedule was used for both the individual and focus group interviews. The schedule was reviewed by some of the research team after the first round of interviews and required no major changes. Two members of the research team (RM and JB), who were not well known to the participants, collected the data between late 2018 and 2019. Both interviewers were female; one was a postdoctoral researcher with extensive experience in qualitative research and the other had an academic education in psychology.

### Recruitment

Using purposive sampling participants were recruited from 14 mental health services involved in delivering the intervention, ensuring a geographic spread, mix of health care areas, and urban and rural representation. Based on their ability to inform the study objectives, participants were selected from different groups of stakeholders and different sites. For recruitment, potential participants who had previously provided consent to be contacted by the EOLAS project team were sent a letter of invitation and a participant information leaflet (PIL), together with an invitation to contact a member of the research team if they were willing to participate, in either an individual or focus group interview. The PIL contained aims of the research and information on data collection and consent process. Once a potential participant made contact all questions were answered and a time for either an individual or focus group interview was arranged. Interviews were either conducted face-to-face or by phone, depending on participants’ preferences, and took place in a hotel, mental health service or university. Participants were required to provide written and verbal consent for the interviews. In addition, the principle of process consent was applied [42], thus the researchers sought verbal consent throughout each encounter. Participants were also informed that they could review the transcripts if they so wished, no participant took up the offer. Interview duration varied between approximately 30 min to one and a half hours. Fieldnotes were recorded after each interview.

### Data analysis

Interviews were transcribed and the transcripts were cleaned to remove any identifying information. All data transcripts were managed and analysed using NVivo version 12 [43] with fieldnotes being used to inform context and support interpretations. Using framework analysis data were systematically coded to each of the domains and constructs of the CFIR framework [35]. The coding process moved through several iterative coding phases, paying particular attention to the ‘goodness of fit’ [44] between the data and the relevant construct and its definition within each domain. To enhance the rigour of the qualitative analysis, data were analysed independently by more than one person (RM, CD and AH) and findings compared. While definitions of each domain and construct within that domain was agreed prior to analysis, the authors acknowledge that in reality some of the constructs overlapped, which required discussion and agreement by consensus, to ensure consistency in data interpretation and avoid overlap in the final write up. Merging of data from different sources was feasible, allowing findings to emerge across all domains; however, some constructs within the domains did not have any supporting data. The frequency of data recurrence and the absence of no new information, indicated that data saturation was reached.

## Results

### Participant characteristics

In total, exceeding the team’s anticipated target, 75 people participated in the study, 42 in one-to-one interviews and 33 in focus group interviews. Participants included EOLAS co-ordinators (*n* = 16), EOLAS facilitators (clinical *n* = 12; peer *n* = 25), programme participants (*n* = 16) and other key stakeholders (*n* = 6). The stakeholders consisted of EOLAS Steering Committee members (*n* = 3) and project workers (*n* = 3) who had been employed to support the development and roll out of the programme. A breakdown of the profile of interviewees is given in Table 2.

**Table 2:** Overview of interviewees by method of data collection and role in EOLAS

Role in EOLAS	Interviews	Demographic Information
EOLAS Clinical Facilitators Nurse = 6; Social Worker = 4; Occupational Therapist = 2	Total = 12 Individual interview = 2 Focus group = 10	*Gender:* Female = 10; Male = 2 *Years working in Mental Health Service:* Mean = 15.58, SD = 8.93 *Years involved with EOLAS:* Mean = 4.5, SD = 2.38 *Number of EOLAS programmes delivered:* Mean = 3, SD = 1.83
EOLAS Coordinators Nurse = 8; Social Worker = 7; Psychiatrist = 1 (12 had experience of facilitating the EOLAS programmes)	Total = 16 Individual interview = 7 Focus group = 9	*Gender:* Female = 11; Male = 5 *Years working in Mental Health Service:* Mean = 16, SD = 11 years *Years involved with EOLAS:* Mean = 3.43, SD = 1.89
EOLAS Peer Facilitators Family Member = 11; Service User = 14	Total = 25 Individual interview = 11 Focus group = 14	*Gender:* Female = 15; Male = 8 Not reported =2 *Number of EOLAS programmes delivered:* Mean = 2.29 *Number currently facilitating an EOLAS programme: n* = 18
EOLAS Participants Family Member = 12; Service User = 4	Total = 16 Individual interview = 16	*Gender:* Female = 11; Male =5 *Length of time since EOLAS completion:* < 1 year = 8, > 1 year = 7
Other Key Stakeholders EOLAS Steering Group members = 3; Project Workers (former and current) = 3	Total = 6 Individual interview = 6	*Gender:* Female = 2; Male = 4

### Enablers and barriers of implementation based on CFIR

**Fig. 1:**
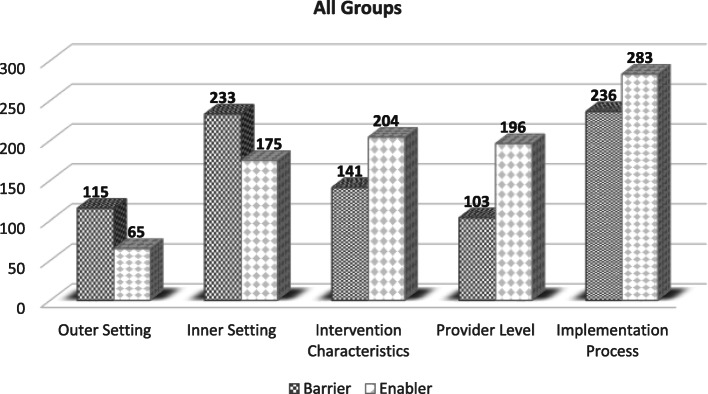
Number of enabler and barrier codes per domain

Participants described a highly complex range of factors that mapped to the five CFIR domains. While some groups of participants spoke in greater depth to a specific domain, all groups identified issues across all five domains, with the exception of the programme participant group who mainly contributed to the ‘intervention characteristics’ domain. In addition, some domains yielded a greater number of coded units (pieces of data) in comparison to others, with the breakdown between barriers and enablers also differing. For example, the implementation process domain yielded 519 code units whereas only 180 pieces of data were coded to the outer setting. Similarly, there was variation in numbers when data were coded as an enabler or a barrier. With regard to the outer setting there were 115 coded references (units) made to barriers and 65 made to facilitators, with many comments referring to the same construct or idea. Figure 1 provides a breakdown of the number of enabler and barrier coded units per domain.

**Fig. 2:**
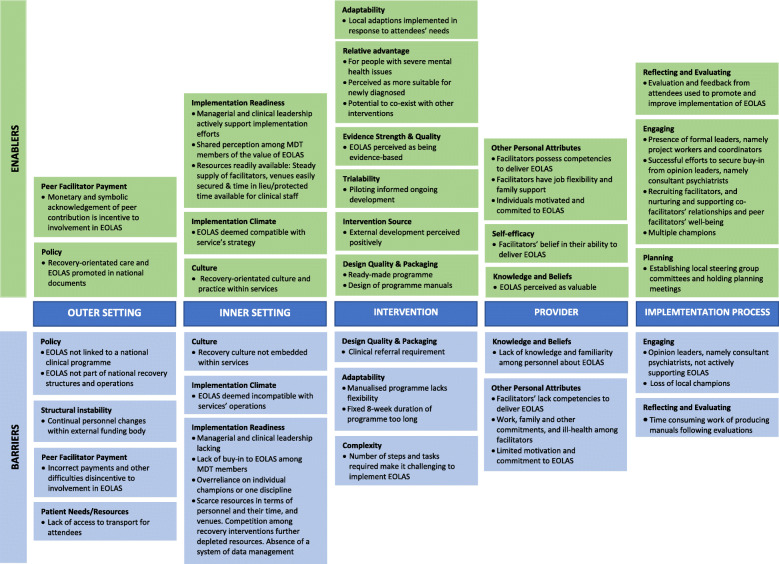
Summary of barriers and enablers

Analysis of the data identified enablers and barriers across all five CFIR domains, with some of the factors identified as being both an enabler and a barrier depending on context. Figure 2 provides an overview of the findings per domain and Additional File 6 provides data to support findings corresponding to the numbering used in each domain.

#### Domain A: outer setting

The Outer Setting focuses on the socio-cultural and infrastructural context in which an organisation resides [35]. Within this domain four key factors were identified: policy, instability intrinsic to the statutory agency, payment, and infrastructure.

In terms of policy there were mixed views, with some participants expressing the view that EOLAS’ compatibility with the national recovery agenda, and the fact that it was referenced in national mental health documents were an endorsement which gave the programme legitimacy and facilitated its promotion within local services (A1-A2: file 6). In addition, some contributors viewed the independence of EOLAS as critical to its success, being developed and co-ordinated by a steering group independent of the Health Service Executive (HSE), and ensuring ongoing fidelity to the EOLAS model (e.g. in training, co-production and co-delivery). This group expressed concern that full assimilation of EOLAS into HSE structures would result in a loss of fidelity to its ethos (A3-A4). On the other hand, other contributors commented on the fact that the external positioning of EOLAS potentially led some stakeholders to consider it to be an ‘addendum’ programme and made the EOLAS Programmes more vulnerable if choices were to be made regarding funding (A5-A8).

The second factor identified as a barrier to implementation related to the structural and personnel instability intrinsic to the HSE who were the funders of the programme. Senior personnel within the HSE who were key to supporting the implementation transitioned out of roles and as a consequence the EOLAS steering group were constantly building and renewing relational ties with funding decision makers and educating them about the unique features and benefits of the programme (A9-A10).

The third factor identified was the payment of peer facilitators. All participants agreed that payment of peers was an important enabler as it valued their contribution and ensured they were not out of pocket; however, they were adamant that the model of payment operated by the health service for peer delivered programmes (including EOLAS) was a significant barrier. In their view it was causing significant distress to some service users and family peer facilitators, due to its unwieldy nature, and as a consequence had adversely affected social welfare payments, (including cuts to social welfare and other entitlements or incorrect and untimely payments) and added to the workload of local co-ordinators (A11-A14).

The fourth factor impacting implementation related to the infrastructural context in which the mental health service operates. The lack of public transport, particularly in rural areas, was identified as a barrier to people accessing EOLAS (A15-A16). Clinical facilitators and coordinators emphasised the importance of taking people’s transport needs and their fears of going out in the evenings into account when arranging the location and timing of the programmes (A17-A19).

### Domain B: inner setting

Inner Setting is defined as the internal socio-cultural context of the organization in which an innovation is being implemented (e.g. cultural, leadership, values, innovation climate, organisational capacity) [35]. Within this domain the following issues were identified: culture, implementation climate and readiness for implementation.

From a cultural perspective, the degree to which a recovery approach to mental health service provision was embedded within the organisational culture and practice was viewed as a critical factor (B1-B2: file 6). As co-production and co-facilitation represented a significant cultural shift, where services were promoting service user participation there was a greater openness and willingness to implement the programme (B3-B4). In contrast, in those services that had not fully integrated recovery principles, and where the medical model was perceived to dominate, the pace of implementation was slower (B5-B8).

In terms of implementation climate, there were mixed views on the degree to which the programme’s alignment and compatibility with the principles and values of recovery facilitated its acceptance and smooth integration into work plans. Some people believed EOLAS was an efficient way to fulfil the organisation’s local commitments to delivering recovery-oriented care (B9-B10). However, others considered that its recovery orientation was a barrier in some settings, such as acute inpatient care. In this context EOLAS was not viewed as a natural fit, as the emphasis within such services was on containment rather than recovery (B11).

In terms of implementation readiness, there was a consensus that organizational leadership and resources were critical. In services where senior nursing and medical personnel proactively promoted the EOLAS programmes, this greatly facilitated implementation (B12-B13). These key stakeholders not only leveraged their managerial position to promote integration of EOLAS into services’ plans and operational processes (B14-B15), but they enabled information about the programmes to be transmitted through services, increasing buy-in at local level (B16). In addition, they adopted a flexible approach to time management, which facilitated clinical staff to manage the demands of their workload, while contributing to EOLAS implementation (e.g. time in lieu) (B17). In contrast, where there were difficulties in engaging the support of senior clinicians (nursing, medical), programmes were delivered in a more ad hoc manner and not fully embedded within services (B18-B19). Similarly, where there was a consensus among Multidisciplinary team (MDT) members around the importance and value of the programme, support for implementation was achieved across teams (B20-B21). However, implementation was hindered in teams were there was a lack of buy-in among team members (B22), and particularly consultant psychiatrists (B23), or where there was an over-reliance on one discipline (nursing, social work) or individual champions to implement the programme (B24-B25). Where this occurred, there were significant challenges in generating referrals and enlisting MDT members as guest speakers.

Another aspect of implementation readiness is resources. In terms of resources, where few competing programmes existed, the allocation of resources to support the delivery of EOLAS was straightforward (B26). However, as the number of recovery-oriented programmes increased, competition between programmes emerged. This decreased the availability of support, and personnel to EOLAS, including potential programme participants, as many of the same services users/family members were being targeted for participation in other programmes (e.g. Behavioural Family Therapy, WRAP, Early Intervention in Psychosis Program) (B27-B28).

The level of human resources dedicated to implementation was also an issue. In some services a surplus of facilitators existed which enabled services to accommodate unexpected facilitator absences (B29-B30). Other services experienced difficulties because facilitators (clinical and peer) dropped out due to changes in circumstances (B31-B32). In addition, the availability of time was consistently reported to be a key factor influencing implementation. Some were of the view that their co-ordination or facilitation roles didn’t impact greatly on their current workload, either because the programme operated outside of clinical hours or they had a sufficient degree of flexibility within their roles to enable them to manage their schedules, including receiving time in lieu (B33-B34). In contrast, others reported difficulty in finding adequate time for the planning and preparation required by the programme, either due to the lack of protected time or because the role was considered an add-on to an already cumbersome workload (B35-B36). In similar vein, some reported challenges in securing protected time for staff to attend the EOLAS training programme.

Some services provided venues from within their existing room complement, whereas others, who wished to run the programme in a community setting, experienced difficulties securing venues (B37-B38). The final resource issue was an absence of a system of data management, which prevented teams from being able to systematically identify individuals and families who might be eligible to participate in EOLAS (B39-B40).

### Domain C: intervention characteristics

The intervention domain focuses on aspects of the intervention. Within this domain six key factors were identified: design; evidence strength; relative advantage; trialability; adaptability; and complexity.

In terms of design, participants reported that the ‘ready-made’ manualised programme was an important enabler for a number of reasons. Where participants perceived they had deficits in service provision or had limited resources to develop programmes to respond to the needs of people with severe mental health problems and/or family members they reported finding the ‘ready-made’ nature of the programme appealing (C1-C3: file 6). In addition to the handbooks being perceived as well-written (using layman’s language) and user friendly (easy to read and follow) (C4-C5), they were deemed to be a comprehensive source of information for service users and family members (C6-C7). The Facilitators Handbooks were viewed as providing structure, support and guidance on delivering the programme as well as aiding facilitators to open communication with participants (C8-C11). While the handbooks were appraised as effectively bypassing the time and resource challenges participants encountered when trying to establish similar initiatives, there were mixed views around the referral aspect of the design. Participants understood the rationale for referral through the Multi-Disciplinary Team (MDT) for example to ensure that only people with the relevant diagnoses were referred and that participants had ready access to support from the MDT if needed (C12-C13). However, some contributors felt that the referral pathway (through Community Mental Health Teams) contravened the ethos of recovery, on the basis that all recovery education needs to be embedded within community facing initiatives, such as recovery colleges (C14-C16). In addition, some perceived the referral pathway as limiting the opportunity to advertise and promote the programme outside of local services, in-turn impeding recruitment of sufficient numbers to sustain the programme on an ongoing basis (C17-C18).

Participants noted that the information within the handbooks was strongly evidence-based, which enhanced their legitimacy and credibility and buy-in within services (C19-C20), with many viewing the piloting and evaluation of the programmes as an enabler. In their view, these ensured that key informants were consulted about the content, structure and delivery of the programme and the information gleaned in turn informed the ongoing development (C21).

The programmes were also perceived to have a number of relative advantages compared to other interventions, including filling an educational void in relation to psychosis and severe mental illness (C22); being suitable for people recently diagnosed and starting their recovery journey (C23); and having the potential to run alongside other interventions (e.g. Behavioural Family Therapy, WRAP), thus providing service users with a stepped pathway to recovery (C24). In terms of adaptability, there were mixed views. While some facilitators were of the view that by its nature, a manualised programme was rigid and lacked a certain amount of flexibility (C25-C26), most were of the view that the programmes offered a fair degree of flexibility which enabled them to be responsive to the needs of programme participants (C27-C28) as well as enabling them to harness different theoretical perspectives (C29). Some were of the view that the duration of the programme acted as a barrier, with the 8 weeks being a considerable commitment for participants (C30-C31).

The extent of work involved in organising programmes acted as a barrier, as participants perceived it as a complex intervention to implement. Co-ordinators recounted the numerous tasks that had to be fulfilled in order to advance implementation, including recruiting and training facilitators, relationship building with and between facilitators, securing venues and guest speakers, promotion and awareness raising amongst their colleagues, prompting colleagues to refer, assisting with payment difficulties, assisting with the research evaluation and reporting progress back to managers (C32-C33). Although each task in isolation was not particularly onerous, cumulatively they were time-consuming in the context of coordinators and facilitators existing workload (C34-C35).

### Domain D: characteristics of individuals (provider level)

The characteristics of individuals or provider domain is defined as ‘Aspects of the individual provider who implements the innovation with a patient or client’. In the context of EOLAS this included both the clinical and peer facilitators. Within this domain three key factors were identified: Beliefs about the Intervention; Self-efficacy; and Other Personal Attributes.

While some participants were of the view that some members of the mental health teams still lacked knowledge about the programme which impeded their ability to promote it (D1-D2: file 6), participants themselves highlighted the need for such programmes (D3-D5) and had a belief in their own ability (self-efficacy) to deliver them (D6-D7). Facilitators’ self-efficacy was attributed to the Facilitator training programme, regular practice, and prior facilitation experience or clinical experience (D8-D10). In terms of other personal attributes, facilitators were of the view that having the ability to communicate compassionately and engage group programme participants, manage group dynamics, manage dual identities (as facilitator and as clinician/peer), negotiate potential tension between content delivery and time constraints, and navigate a non-hierarchical co-facilitation relationship, were important for effective facilitation (D11-D14).

While some facilitators felt they successfully expressed these competencies, others recounted challenges. Clinical facilitators recalled struggling with letting go of their ‘expert’ status and hierarchical position (D15), while some peer facilitators reported struggling with managing their dual identity of co-facilitator and service user, and in establishing an equal partnership with clinical co-facilitators (D16-D17). Job flexibility and family support enabled some family facilitators to participate (D18-D19), while conflicting commitments and ill-health limited the availability of some service user facilitators (D20-D21). Finally, participants considered that a key enabler was the personal motivation and commitment of each individual involved. Facilitators who were motivated and committed were deemed to continuously promote the programme, follow-up on recruitment efforts, engage with and support potential programme participants and make themselves available to deliver the programme when needed (D22-D24). However, some participants noted that motivation was limited to a core group of dedicated individuals (D25-D26).

### Domain E: implementation process

In terms of implementation key factors identified included planning, engaging key stakeholders and champions, and evaluation.

In terms of planning, identifying formal leaders was of key importance. The hiring of paid project workers, to coordinate the overall project and the establishment of local steering groups within services was viewed as critical. The role of the project worker was not only critical in introducing services to EOLAS and encouraging them to adopt it, but they provided ongoing support to co-ordinators and facilitators on day to day issues (E1-E2: file 6). Having a local steering group that comprised all stakeholders was also central, as this group mapped the local pathway to rolling out EOLAS and addressed issues and concerns related to resources, recruitment, and promotion (E3-E5). Many services also appointed a coordinator, from within existing resources, to oversee local implementation, which meant the person took on the extra responsibility of coordinating EOLAS. Having a coordinator with status, credibility and who was capable of influencing and persuading key influential people (E6-E7), was vital to getting EOLAS off the ground. While the coordinators’ activities varied from service to service, coordinators who linked with, and supported clinical facilitators was a key enabler (E8-E9), as successful implementation depended on them working together to plan advertising, dates, venues, guest speakers, and secure and follow up on programme participant referrals (E10-E11).

Successful implementation also involved recruiting and engaging key stakeholders, such as consultant psychiatrists. Securing buy-in from consultant psychiatrists was critical, as they were perceived as a powerful group with significant influence on a team’s approach to care (E12-E13). Coordinators and clinical facilitators spoke of using a number of strategies to engage this group, including presenting evidence from evaluations to having family members and service users make presentations about EOLAS at medical fora (E14-E15). When support (beyond verbal tokenism) was not achieved, referrals to the programme were not forthcoming (E16-E17). In addition to consultant psychiatrists, the recruitment of facilitators was considered critical. While recruitment of clinical facilitators was through word of mouth within services, recruitment of peer facilitators was more challenging. Factors that supported recruitment of peer facilitators, included clinicians having well established connections within community settings and knowing which service users and family members might be interested in becoming a facilitator (E18-E19).

From a peer perspective, a key enabler was the credibility and nature of the interaction they had with the clinician or project worker who issued the invitation (E20-E21). Once recruited and trained, part of successful implementation involved co-facilitators (and sometimes coordinators) meeting prior to and after each session, to plan sessions and foster collaborative, non-hierarchical working relationships (E22-E24). Clinical facilitators and coordinators also described the importance of supporting the wellbeing of peer facilitators (E25-E26), as well as ensuring that all trained peers got opportunities to facilitate programmes (E27).

Successful implementation was also attributed to having multiple dedicated and active local champions (coordinators, clinical facilitators, mental health nurses) within teams who constantly kept EOLAS on the agenda by promoting it at meetings, and continually engaging and following up with colleagues to create an awareness and understanding of EOLAS and to increase referrals (E28-E30). In contrast, in services that depended on a single champion, the loss of this person through turnover or role change threatened implementation (E31-E32) and succession planning for staff turnover was felt to be needed (E32-E33).

The formal evaluation and feedback processes that was built into the EOLAS process were also perceived as an enabler, as this enhanced buy-in among clinical and management personnel (E34-E35), and enabled participants to identify ways in which EOLAS implementation could be improved (E36). While the evaluation process was an enabler, the time-consuming nature of producing and updating the programme handbooks was an unanticipated barrier, as delays in the availability of up-to-date handbooks slowed down implementation within sites for a time (E37-E38).

## Discussion

The purpose of this study was to explore the factors mediating the implementation of recovery-oriented psychoeducation interventions. Using the Consolidation Framework for Implementation Research (CFIR) [35], key enablers and barriers were identified across all five domains, with some factors (depending on context) being both an enabler and a barrier. To our knowledge, this is the first study that used an implementation theory to explore factors influencing implementation of a group psychoeducation intervention.

Introducing a new intervention into a mental health service is not just a local phenomenon [45], but an endeavour that taps into the complex relations that exists between the healthcare system and the wider outside world [46]. To date, examination of determinants (such as mental health policy and national health system governance) on the adoption and implementation of psychoeducation is limited. In this study, the epistemological alignment of EOLAS to national recovery-oriented mental health policy [47, 48] and other HSE recovery documents [49, 50] proved to be a strong enabling factor to local service adoption, as local decision makers perceived EOLAS to be apt model for the effective integration of recovery-oriented care into local service provision.

While the presence of a National policy on recovery [47, 48] has a major influence on programmes such as EOLAS, policy alignment is not always sufficient, as policy is constantly evolving in response to changing societal and political expectations. In addition to this natural evolution in social and political priorities, the Health Service Executive (the body charged with the implementation of national health policy) has also been subject to recurring phases of structural and personnel change (e.g. moving from the delivery of mental health services through a National Division for Mental Health, to new localized structures). This flux in health service structures and personnel, as identified by participants in this study, also added significant challenges, such as the need for repeated engagement to build relationships of trust and credibility with newly appointed decision-makers who controlled the allocation of financial resources.

In addition, the nature of the governance of the programme was also an issue as it was perceived to be independent of national and local service structures. Whilst independent governance has advantages, such as enhanced control of fidelity, it can introduce vulnerability, particularly when competing initiatives emerge which are fully integrated into national as well as local governance structures. In this landscape, an independently governed intervention such as EOLAS can ultimately be perceived as an ‘optional extra’ to service provision and consequently experience challenges in securing financial and personnel prioritization from senior health service managers. This finding highlights some of the challenges in achieving a balance between independent governance and sustainable integration of an intervention within existing statutory services, an area that has received scant attention in the literature and is worthy of further study. Of note, the independent governance of some programmes, such as Behavioural Family Therapy (BFT) and Wellness Recovery Action Plan (WRAP), both located outside of Ireland, do not appear to cause similar concerns for contributors. The reason for this disparity in organisational attitudes to domestic programmes as distinct from programmes originating overseas is unclear.

While these findings highlight how factors in the outer domain of policy influenced adoption, within the inner setting, the importance of strong and credible leadership across all levels of the mental health service to the implementation of EOLAS emerged as a central theme. This leadership helped to successfully negotiate inter-organizational relationships and supported the cultural shift towards recovery and, by extension, inclusion of co-production and co-facilitation strategies. Where influential and engaged leaders existed at senior level within organisations, other key stakeholders were brought on board to support and deliver the programmes; champions of the programmes were facilitated at lower levels, and resources (including flexible work practices) were made available. This lends credence to findings from other studies who report on the centrality of synergistic leadership and managerial support across mental health services when implementing psychoeducation programmes [51–53]. It also highlights how the success or failure of implementation can rest on leaders’ ability to navigate vertical and horizontal inter-organizational relationships [54, 55].

The implementation of any complex intervention and transition from initial adoption to routine practice is “a nonlinear process characterized by setbacks and unanticipated events” [[13] p. 610]. Similar to other studies, the efficacy of the intervention was cited to be a critical facilitator to implementation [34]. The intervention’s rigorous development, piloted feasibility, tested efficacy, and evidence-based handbooks convinced many stakeholders of its value. Of equal importance was the intervention’s ability to integrate harmoniously with ongoing efforts to develop a recovery focussed ethos within local services, which supported its adoption.

However, there were elements of the EOLAS programme structure that were felt to hinder implementation, such as the referral process. The challenges of securing referrals and the importance of engaging continually with clinician providers, especially consultant psychiatrists, to normalise the referral process within work practices has also been a challenge in other programmes [52, 56, 57]. Contributors in this and other similar studies report that some consultant psychiatrists harnessed their position as team leaders to support multidisciplinary teams to adopt recovery-oriented approaches such as EOLAS. On the other hand, where the psychiatrist was not on-board, obstacles ensued. In this regard, the recent publication of the Position Paper on recovery published by the College of Psychiatrists of Ireland in 2020 [58] is welcome, as not only does it outline how psychiatrists can embrace recovery principles but highlights the role of the EOLAS Programmes (e.g. including a psychiatrist as a ‘guest speaker’) as an example of the collaborative approach that is fundamental to recovery, and one that supports the diffusion of recovery principles in clinical practice [59].

Consistent with other studies that have explored barriers to implementation of group psychoeducation, issues such as access to resources (e.g. of time and venues), operation outside existing work patterns/shifts or over-reliance on a single ‘champion’ contributed to the difficulties in local implementation [53, 60, 61]. While these are not unique or specific to the EOLAS programmes and reflect systemic and structural challenges within health services more generally, they once again highlight the importance of strong leadership and adequate resourcing within mental health services to address these issues.

The study findings also bring into sharp focus the supports required to implement a co-produced and co-facilitated intervention. Despite the positive appraisal of the facilitator training, both clinical and peer facilitators noted challenges in developing genuinely equitable partnerships in practice, revealing that at times they slipped back into their traditional hierarchical roles of ‘patient’ and ‘provider’. Given that the implementation of co-production is in its infancy in mental health services [62–65], this is not a surprising finding and highlights the importance of support during intervention implementation (in the form of informal mentoring or clinical supervision), as well as endorsement by service leaders and senior managers. In the context of coproduction, the availability of a streamlined and hassle-free payment system for peers is of critical importance. Study findings indicate that many peer facilitators experienced challenges around payments. If we are to meaningfully enact parity of esteem between service users and clinicians, the necessary bureaucratic infrastructure needed to achieve it must be firmly embedded. Failure to do so risks imposing undue stress and break down of trust between peers and services, alongside the potential attrition of peer partners. An additional concern identified in the inner domain is the reliance on a small cohort of ‘champions’ (mostly from the disciplines of nursing and social work) to drive the implementation of EOLAS locally. This highlights the importance of viewing the implementation of change as a ‘whole team’ challenge, where each discipline and individual shares the responsibility to actively seek out and implement recovery oriented programmes such as EOLAS, rather than allowing this responsibility to be ‘siloed’ to a particular discipline (such as nursing). Where responsibility is widely shared across the MDT, it is more likely that the experiential learning arising from implementation and the move to recovery orientated practices will be enhanced**.**

Equally, the findings highlight the challenges of overcoming local interpretation of national policy in relation to recovery. In some services, recovery-oriented care was viewed by contributors as situated in and facilitated exclusively through the community, for example as something that must be underpinned by the principal of self-referral, and not overly influenced by the “medical model”. Consequently, some staff appraised EOLAS as not ‘fitting’ with their view of recovery-oriented care. This finding demonstrates that for optimal adoption into routine clinical practice there is a need to move beyond the narrow lens of simply emphasising the value and efficacy of the intervention, towards understanding views, preferences, needs, or demands of potential adopters [12]. This finding is also important, in that the more narrow concept of recovery has the potential to leave people who are at an early phase of their recovery journey without critical information necessary to engage positively with mental health services and has the potential to sideline those who do not have the self-confidence to self-refer to community based resources. These views also continue, perhaps inadvertently, to perpetuate the positioning of the recovery perspective in opposition to psychiatry [66, 67]. A discourse suggesting it is either/or as opposed to both/and, which is a perspective that possibly closes down debate as opposed to exploring contradictions with an openness to new possibilities and perspectives.

### Limitations

The CFIR, which informed data collection and data analysis, helped capture both the breadth and depth of implementation determinants and ensured that they were systematically captured and appraised across all domains, thus strengthening the efficiency, generalisability, and interpretability of the findings. In addition to helping the authors to tell a story in an organised and comprehensive manner, by using the CFIR framework and its underpinning constructs, we have contributed to an implementation science evidence base and enabled future researchers to replicate and compare findings from other studies and contexts. However, using the CFIR is not without challenges. Capturing enough depth and specificity across all CFIR constructs within the time limitation of a single interview is difficult to achieve. As a result, nuanced data within a construct, which may be critical to implementation, may have been missed. The number of constructs within each domain also induced complexity during data analysis, particularly in the write up as some constructs appeared to overlap, suggesting a need for further exploration of the CFIR. In addition, further studies are required to identify the most relevant CFIR constructs required for successful implementation as well as longitudinal studies into how an implementation process evolves over time.

While the inclusion of multiple sites and different cohorts of stakeholders provided a triangulated and deepened understanding of factors in real-world settings [10, 68–70] and thus addressed some of the methodological issues identified in previous studies [34], there are several limitations that need to be acknowledged. First the self-selection nature of the sample may have resulted in recruitment bias, with those who were more assertive, confident, and articulate and with strong views opting to participate. As a result, it cannot be assumed that the views and experiences presented represent all of those who were involved in the implementation process. Secondly, the focus of recruitment was on participants directly involved with the programme, thus other opinion leaders such as service managers, consultant psychiatrists or Directors of Nursing not directly involved or those tasked with developing other recovery-oriented initiatives were not involved. The findings are also a result of interviews as opposed to observation of practice, and may be influenced by both recall and social desirability bias. In addition, while the researchers endeavored to minimise interpretative bias during the analysis by using the constructs underpinning CFIR and by having more than one person complete the data analysis, there is always the potential for misinterpretation as qualitative data analysis has a subjective element.

## Conclusion

The implementation of recovery-oriented change faces considerable challenges and obstacles to sustainability. Change theorists advocate for comprehensive pre-implementation planning, including adequate consideration of how the intervention can be delivered with high fidelity whilst also harmoniously integrating it into existing systems, structures, and workflows. Findings from this study provide an enhanced understanding of the different layers of determinants to implementation of a recovery focused psychoeducation intervention across the range of domains and help illuminate why setbacks may occur. Overcoming these challenges will involve positive and ongoing engagement and collaboration across the full range of stakeholders that are active within each domain. The quality of leadership at each domain level is of crucial importance to a successful outcome, including at MDT level, Mental Health Engagement, Service User fora and within professional bodies for mental health disciplines. However, health service management at both policy and operational level have a particular responsibility to find ways of positive engagement with all stakeholders in developing effective implementation strategies that truly respect the value of collaboration and partnership in achieving positive change.

## Supplementary Information

**Additional file 1.**

**Additional file 2.**

**Additional file 3.**

**Additional file 4.**

**Additional file 5.**

**Additional file 6.**

## Data Availability

The datasets generated and analysed during the current study are not publicly available due to privacy concerns but are available from the corresponding author on reasonable request.

## Abbreviations

CFIR: Consolidation framework for implementation research
MHS: Mental health systems
WRAP: Wellness recovery action plans
IMR: Illness management and recovery programs
HSE: Health service executive
MDT: Multidisciplinary team
BFT: Behavioural family therapy (BFT)
