# Supplementary material for: Factors impacting the implementation of a psychoeducation intervention within the mental health system: a multisite study using the consolidation framework for implementation research
Source: BMC Health Serv Res. 2020 Nov 9;20:1023. doi: 10.1186/s12913-020-05852-9 (PMC7654573; doi:10.1186/s12913-020-05852-9)
Supplement: Supplementary file 1 — Additional file 1. [file 12913_2020_5852_MOESM1_ESM.doc]

**Semi-structured Interview Guide for EOLAS Stakeholders**

**Opening questions**

- Some general background information from Interviewee.
- Could you please describe what your role/involvement in EOLAS is/was?

**Historical Implementation (1^st^ roll out into other services)**

Can you tell us about the processes/negotiations/steps involved in the early days of trying to establish EOLAS into services?

What challenges did you experience in those early days?

What do you believe were the factors which began ‘to make a difference’ in getting EOLAS into other services?

**Organisational – Local Factors**

- Can you tell us about the processes/steps which services need to complete in order to begin delivering EOLAS?
- Can you identify factors which often **hinder service’s** initial adoption and delivering of EOLAS? and how are these challenging factors minimised or resolved?
- Can you describe factors which **facilitate** the initial adoption and delivering of EOLAS?
- Are there any other systemic/cultural/structural/personnel/intervention factors which facilitated/challenged the implementation of EOLAS? Please describe.

**Organisational - National Factors**

- In your experience, is there commonality in the challenges that services experience or are there also often site-specific characteristics, which need to be considered?
- Can you tell us how broader national factors may influence/d services’ adoption and implementation of EOLAS? (prompts – e.g. HSE management structures/dynamics, national mental health policy, funding structures)

**Sustainability –**

- Are there systemic/cultural/structural/personnel/intervention factors which continue to challenge the implementation of EOLAS? Please describe.
- What do you believe are the factors necessary to ensure the sustainability of EOLAS?
- Are there ways you think that EOLAS could be further integrated/embedded into the mental health service?

**Impact**

- In your experience, what have been the long-term benefits of the EOLAS programme?
- Do you think EOLAS has had an impact on systemic culture and practice in the wider mental health team and service?
- Are there aspects of EOLAS which you think are easier/more difficult to incorporate into wider clinical practice than other aspects?
- Ways you think that EOLAS could strengthen long-term outcomes for service users/family members/clincians/mental health team/mental health service?

**Conclusion to the interview:**

****Make sure to ask the participant before concluding interview if they have anything further to add on any of the issues raised, or perhaps they felt that certain topics were not included in discussion.****

- Explain how/when/where interview data will be stored and disposed of
- Answer any questions the participant raises
- Remind the participant that a copy of the interview transcript will be sent to them if they wish
- Check participant well-being
- Thank participant for their involvement
