# Supplementary material for: Factors impacting the implementation of a psychoeducation intervention within the mental health system: a multisite study using the consolidation framework for implementation research
Source: BMC Health Serv Res. 2020 Nov 9;20:1023. doi: 10.1186/s12913-020-05852-9 (PMC7654573; doi:10.1186/s12913-020-05852-9)
Supplement: Supplementary file 2 — Additional file 2. [file 12913_2020_5852_MOESM2_ESM.doc]

**Semi-structured Interview/Focus Group Guide for EOLAS Co-ordinators**

**Opening questions**

- Some general background information from Interviewee.
- Could you please describe what your role/involvement in EOLAS is/was?

**Exploratory Stage**

- Can you tell us about when you first heard of/encountered EOLAS?
- Can you describe the decision-making process which informed your service’s decision to try to implement EOLAS?
  - Prompts: who was involved in discussions? Can you remember if the Pros and Cons of implementation were discussed? And if so what were they?

**Strategies/actions to implementation**

- Can you tell us about the processes/steps which services need to complete in order to begin delivering EOLAS?
  - Planning, engaging, educating, executing, reflecting and evaluating.
  - Individuals involved, approval processes.

**Challenges to implementation**

- Can you identify factors which **hindered your service’s** initial adoption and delivering of EOLAS?
  - Intervention characteristics (content, delivery, format, recruitment processes, relative benefit)
  - Inner setting (organisational readiness, financial resources, buy-in, leadership, perceived value, relationships, staff cohesion, communication pathways)
  - Provider (skill-set, knowledge, beliefs, adaptability, openness to change, commitment, attitudinal)
  - Service user/ family participant

**Strategies to overcome challenges**

- How were/are these challenging factors minimised or resolved?
  - Prompts: Adapt processes, change personnel, change location, secured support

**Facilitators to implementation**

- Can you describe factors which **facilitated** the initial adoption and delivering of EOLAS?
  - Intervention characteristics (content, delivery, format, recruitment process, relative benefit)
  - Inner setting (organisational readiness, time in lieu arrangements, buy-in, leadership, perceived value, relationships, staff cohesion, communication pathways)
  - Provider (skill-set, knowledge, beliefs, adaptability, openness to change, commitment, attitudinal)
  - Service user/family participant

**Outer Setting - Challenge and/or Facilitate?**

- Can you tell us how broader national factors may have influence/d your services’ adoption and implementation of EOLAS?
  - Prompts – e.g. HSE management structures/dynamics, national mental health policy, funding structures, demand/supply, saturation of market, competition, peer pressure, i.e. keeping up with neighbouring counties

**Sustainability –**

- Are there systemic/cultural/structural factors which continue to challenge the implementation of EOLAS? Please describe.
- What do you believe are the factors necessary to ensure the sustainability of EOLAS?
- Are there ways you think that EOLAS could be further integrated/embedded into the mental health service?

**Impact**

- In your experience, what have been the long-term benefits of the EOLAS programme?
- Do you think EOLAS has had an impact on systemic culture and practice in the wider mental health team and service?
- Are there aspects of EOLAS which you think are easier/more difficult to incorporate into wider clinical practice than other aspects?
- Ways you think that EOLAS could strengthen long-term outcomes for service users/family members/clincians/mental health team/mental health service?

**The future**

- In what ways do you think EOLAS could develop in the future? (prompts e.g. repeated with same cohort, conducted with different cohorts, different settings)

**Conclusion to the interview:**

****Make sure to ask the participant before concluding interview if they have anything further to add on any of the issues raised, or perhaps they felt that certain topics were not included in discussion.****

- Explain how/when/where interview data will be stored and disposed of
- Answer any questions the participant raises
- Remind the participant that a copy of the interview transcript will be sent to them if they wish
- Check participant well-being
- Thank participant for their involvement
