# Supplementary material for: Factors impacting the implementation of a psychoeducation intervention within the mental health system: a multisite study using the consolidation framework for implementation research
Source: BMC Health Serv Res. 2020 Nov 9;20:1023. doi: 10.1186/s12913-020-05852-9 (PMC7654573; doi:10.1186/s12913-020-05852-9)
Supplement: Supplementary file 4 — Additional file 4. [file 12913_2020_5852_MOESM4_ESM.doc]

**Semi-structured Interview/Focus Group Guide for EOLAS Service user/Family Facilitators**

**Opening questions**

- Some general background information from Interviewee.
- Could you please describe what your role/involvement in EOLAS is/was?
- Can you tell us about when you first heard of/encountered EOLAS?

**Challenges to implementation**

- Can you identify factors which **challenged** you in delivering EOLAS?
  - Intervention characteristics (training, manuals, content, delivery, format, recruitment process)
  - Inner setting (payments, buy-in, leadership, perceived value, relationships, dynamic of co-delivery, support, communication pathways)
  - Provider (skill-set, confidence, knowledge, beliefs, adaptability, openness to change, commitment, attitudinal)
  - Service user/family participant

**Strategies to overcome challenges**

- How were/are these challenging factors minimised or resolved?
  - Prompts: Adapt processes, change personnel, change location, secured support

**Facilitators to implementation**

- Can you describe factors which **facilitated** you in delivering EOLAS?
  - Intervention characteristics (training, content, delivery, format, recruitment process, relative benefit)
  - Inner setting (payments, buy-in, leadership, perceived value, relationships, dynamic of co-delivery, support, communication pathways)
  - Provider (skill-set, confidence, knowledge, beliefs, adaptability, openness to change, commitment, attitudinal)
  - Service user/family participant

**Outer Setting - Challenge and/or Facilitate?**

- Can you tell us how broader national factors may have influence/d your services’ adoption and implementation of EOLAS?
  - Prompts – e.g. HSE management structures/dynamics, national mental health policy, funding structures, demand/supply, saturation of market, competition, peer pressure, i.e. keeping up with neighbouring counties

**Impact – personal**

- What do you feel have been the long-term benefits of your involvement in the EOLAS programme?
  - Your Knowledge of recovery approaches, co-production/collaborative working, mental health services, perspectives of clinicians, your recovery/well-being?
  - Your advocacy skills- for your needs, family members needs, for others?
  - Has your involvement in EOLAS changed your relationship you’re your family/clinicians/mental health team in any way?
  - Describe/provide examples of how involvement in EOLAS has impacted upon your interactions with and/or understanding of family members/clinicians/mental health team.
  - Do you feel your involvement in EOLAS has impacted upon your own well-being? If so, in what ways?
  - Have you utilised the skills you learned through EOLAS elsewhere? (e.g employment, education, sitting on committees etc)

**Impact – general**

- Do you think EOLAS has had an impact on systemic culture and practice in the wider mental health team and service?
- Are there aspects of EOLAS which you think are easier/more difficult to incorporate into wider clinical practice than other aspects?
- Ways you think that EOLAS could strengthen long-term outcomes for service users/family members/clinicians/mental health team/mental health service?

**Sustainability –**

- Are there systemic/cultural/structural factors which continue to challenge the implementation of EOLAS? Please describe.
- What do you believe are the factors necessary to ensure the sustainability of EOLAS?
- Are there ways you think that EOLAS could be further integrated/embedded into the mental health service?

**The future**

- In what ways do you think EOLAS could develop in the future? (prompts e.g. repeated with same cohort, conducted with different cohorts, different settings)
