# Supplementary material for: Factors impacting the implementation of a psychoeducation intervention within the mental health system: a multisite study using the consolidation framework for implementation research
Source: BMC Health Serv Res. 2020 Nov 9;20:1023. doi: 10.1186/s12913-020-05852-9 (PMC7654573; doi:10.1186/s12913-020-05852-9)
Supplement: Supplementary file 5 — Additional file 5. [file 12913_2020_5852_MOESM5_ESM.doc]

**Semi-structured Interview Guide**

**(Service Users/ Family Members attendees)**

**Opening questions**

- Some general background information from Interviewee.
- Experience of taking part in the EOLAS programme/ how did you find being part of this programme? /what was it like to take part in this programme?

**Key areas to cover**

- Could you please describe how your attendance at the EOLAS group has impacted on your day to day life?
  - Are the changes you described experienced intermittingly? (e.g. straight after/days after an EOLAS meeting) or are they sustained over a period of time (days, weeks, months)?
- Do you feel your involvement in EOLAS has impacted on your relationships with family members/clinicians/wider mental health team?
  - If yes, can you give examples of how these relationships have changed?
  - Have you experienced other positive benefits as a result of these relationships improving? E.g. improved engagement with service providers? Improved capacity for self-advocacy?
- Has your involvement in EOLAS impacted on your knowledge of severe mental health issues?
  - If yes, in what ways has this helped you?
- Do you feel your perception of yourself and others (both within the group and in the community) has changed in any way? Please describe/give examples.
  - Self-esteem? Self-confidence? Self worth? Sense of belonging? capacity to interact/socialise with others? or try new things (education/work/volunteering)?
- Could you tell us what, if any, impact EOLAS has had on your on well-being and quality of life?
  - E.g. Day to day attitude/outlook? Perceptions of the future? capacity to cope?
- Are there ways you think the EOLAS programme did well or could improve in the future?
  - E.g. thinks that inhibited your participation or helped you to particpate

**Conclusion to the interview:**

****Make sure to ask the participant before concluding interview if they have anything further to add on any of the issues raised, or perhaps they felt that certain topics were not included in discussion.****

- Explain how/when/where interview data will be stored and disposed of
- Answer any questions the participant raises
- Remind the participant that a copy of the interview transcript will be sent to them if they wish
- Check participant well-being

Thank participant for their involvement
