# Supplementary material for: Factors impacting the implementation of a psychoeducation intervention within the mental health system: a multisite study using the consolidation framework for implementation research
Source: BMC Health Serv Res. 2020 Nov 9;20:1023. doi: 10.1186/s12913-020-05852-9 (PMC7654573; doi:10.1186/s12913-020-05852-9)
Supplement: Supplementary file 6 — Additional file 6. [file 12913_2020_5852_MOESM6_ESM.doc]

**Additional File 6: Data to support each domain**

| **Domain A: Outer Setting** | |
| --- | --- |
| **Quote #** | **Participant Quote** |
| A1 | They [National Office] put EOLAS in the service plan, you know. And I suppose at a national level that has made a difference. That we can go into services and say look, we’re part of the service plan. You know EOLAS is part of that. She [Director of the National Office] has promoted us at conferences; you know all of those things make a difference. They give you a legitimacy. SH2 (II) |
| A2 | There has been a general crystallising of a commitment at senior administrative level [in the HSE] towards recovery and recovery framework. And towards that being an important principle in the development and shaping of services into the future […]. And obviously that is very much a facilitator of maintaining the standing of EOLAS with the senior management structures, within the HSE. Because EOLAS fits so well with that ethos. SH1 (II) |
| A3 | There is an argument for the importance of EOLAS staying outside, as from outside, we can ensure its quality framework and training, we can maintain an independent voice of advocacy, we can ensure the primacy of the coproduction with the inclusion of the EOLAS peer facilitator. SH7 (II) |
| A4 | [...] training in here [EOLAS] is not the same as recovery training over here [ARI’s (Advanced Recovery in Ireland) ‘train the trainers’ training]. And that actually the skills aren’t transferable. And if you kind of superimpose one on the other you’ll lose potentially….  **Fidelity integrity.**  All of those things. The core essence of what makes it EOLAS, exactly. And that they don’t transfer. SH1 (II) |
| A5 | I think EOLAS being separate and it remained separate to the actual recovery program within the HSE, began to become more of an issue. Because it was always an add on as oppose to an integral part of. So I think that remains an issue and I think that will also be the downfall of EOLAS unfortunately […] CO5 (II) |
| A6 | And what makes it doubly difficult for EOLAS is […] it's actually outside of the system […]. So I think that outsider piece is very significant to EOLAS in terms of how it actually gets embedded in. And I think there's been an ambivalence about whether there's a wish to have it embedded in or not, all right. SH5 (II) |
| A7 | […] it's [EOLAS] one single project with two part time workers with no staff employed by the HSE. It's vulnerable I think it's slightly isolated and slightly vulnerable no one questions the ethos of it and I think it's very, very strong it's a fairly kind of strong programme but it's not really kind of embedded I think across the HSE the way some of the other strands are. And because it's a single project no HSE staff I think it makes it a little bit vulnerable and I am not sure everybody sees that yes. SH3 (II) |
| A8 | […] it’s [EOLAS] not translating from management team to front line. And I don’t think that’s anything to do with anyone in terms of not wanting to do it, but it’s not under a program. So if it’s not under a program it doesn’t then, on a very basis level it doesn’t then tick any boxes in terms of your operational planning […] CO5 (II) |
| A9 | And a lot of the influencing is done through human relationship. And not through a formal structure… We put a lot of work into establishing the human relationship with important decision makers… getting access {to senior policy people] is not easy …And we had managed to get ourselves invited to a meeting in the national mental health division last year and made a presentation. And it went swimmingly. Then the structure was changed. The person that you’ve been cultivating a relationship with is moved. And all of that work in terms of interpersonal work is gone. Because none of it [learning, relationship] stays behind with the post. Only the structures are left. And essentially you’ve to start all over again. So that’s been a real challenge. ISO1 |
| A10 | We had a meeting with [Director of Mental Health – HSE]. And [names person] learned about EOLAS and gave us our backing. And they [HSE] put EOLAS into the recovery focus, so that was a huge, huge thing , hugely helped, but then the person moves on. … and every year we have to see if it’s going to get the contract [funding], or if it’s going to be renewed. IS06 |
| A11 | FF6 (FG): So the first say course that we facilitated was this time last year say from September to November. And I got paid just this August gone; I got half of it in July and half in August, or something.  **Q: So it took months.**  FF6 (FG): Yea now I did, I you know I didn’t want to be begging for it. But you know I told my coordinator and she was back and forth the whole time with someone on payroll. |
| A12 | […] there was no information about whether doing this course would affect me disability allowance, social welfare. I was kind of led to believe it probably wouldn’t affect it but then it did affect it. Now I’m at the stage were social welfare are docking at euros off me, it’s very difficult. I’ve put in an appeal and they said it takes months, but it’s been months now since I put in the appeal. SF2 (II) |
| A13 | I’ve had horrendous experience now with a service user co-facilitator now who had their social welfare cut because they did EOLAS and I’ve had to appeal that with them and it’s just been an absolute nightmare to the point where like they’re turned off, and that’s a, that’s a client of mine that I, I really try to mind through the process, I identified as being a very good resource as somebody who would be brilliant, and they were, they were absolutely fantastic through the facilitator process so much and all this additionality has become a major headache, so the likes of that in terms of actually supporting the, the, your co-facilitators and making sure that they’re minded and looked after and that it’s a good experience for them has been hugely problematic. CO15 (FG) |
| A14 | […] it was causing an awful difficult time more so for them, but also for me trying to navigate the system to help them. And at the end of the day I had no background in it [social welfare] and it was really, really disappointing that aspect of it […] CO4 (II) |
| A15 | So I suppose again you come in to a transport issue for people getting from [Large town] up to I suppose where we ran it was [2^nd^ town]. That’s [transport] where we felt the greater need was. So that’s a 40 minute drive and if you’re not driving its 2 buses, so that in itself is a headache. CF1 (II) |
| A16 | And then the challenge of a rural service, you know, getting people in to one location at eight o’clock in the evening on the country roads in November is a challenge I suppose. CO13 (FG) |
| A17 | […] but then transport links in to [large town] – there’s a lot to take on board with it, you know, because we’re conscious of that then when we’re running it [programme], where people are coming from, bus times, school times, holiday times, there’s a lot of stuff. CO1 (II) |
| A18 | I suppose socially and economically in [2nd town in ES07], unemployment is so, so high, access to public transport is very, very poor. So ergo then, if your initiatives are very centralised in [Large town in ES07] or [City in Southern Ireland] it’s very hard for people to access anything. So I suppose that was my own personal agenda to get something running in [2nd town in ES07] that people can access easily on their doorstep. CF1 (II) |
| A19 | Yea and it just can be hard to, also to get the timing right. Like so do you run it in the day time, do you run it in the night time? Like people are working during the day. So it’s better to run it at night time. But then at the same time it can be hard to get travel you know, to get buses and the dark in the evening, the weather, all of that. So there’s a lot of especially in the country, where there isn’t that kind of availability of transport you know. CO8 (FG) |
| **Domain B: Inner Setting** | |
| B1 | I was just going to say I think it depends on how lucky you are in your area if it is a recovery culture and if your consultant buys in, if you’re team is recovery focused […] CF9 (FG) |
| B2 | It depends on how I suppose to be honest how convinced they [senior management] are about the principles of recovery because it's a shift away from just a clinical or kind of medical approach. The approach of having people at the centre of their own recovery and the ethos of EOLAS is that they operate as equal partners in the training and then the delivery of local programmes. SH3 (II) |
| B3 | […] it wasn’t the traditional sort of service. It was very outward looking and recovery focused sort of service, you know what I mean. So that’s where the initial programmes were launched, was in [ES12] SH6 (II) |
| B4 | I think in that sense this whole idea of co-facilitation, there was something kind of a little scary about it at the start and […] people are fine with it now because they are so used to it, whereas I think at the start everyone was like oh, I don’t, you know, it was, you hadn’t done it before and they were, we were like I don’t even know what I would say, […] it’s just because it was, really we came, you know, like we came from a completely institutionalised hospital based service out to this whole, you know, we were thrown in to this recovery model and it was just such a huge change and it was, it took a while but - it actually didn’t take that long – I think people adapted really quickly, once it started coming in to the service people were fine with it and, you know  CO1 (II) |
| B5 | No because our team definitely it would be more medically led. You know and it would, like EOLAS would be at the very bottom [of priorities]. CF3 (FG) |
| B6 | [...] the medical model is so entrenched that it’s not going to be moved that easily. So any of the recovery work that goes on, it’s almost seen as peripheral or extra. And that is the biggest difficulty [...] SH2 (II) |
| B7 | […] there’s pockets of where it’s, it’s [EOLAS], you know, very accepted and they’re very recovery focused […] and I think it is, there is pockets of a kind of a deeply entrenched cultural attitudes that, that, while they might say they’re recovery focused, the practice says something different […] CO14 (FG) |
| B8 | […] I do hear of other sectors who don’t have a recovery ethos and that can be a barrier [to implementation]. Don’t mess with my patients or come with your new fangled [intervention/approach}.Yeah, it just depends on I think the areas understanding. CF9 (FG) |
| B9 | We were looking at our IMROC [Implementing Recovery through Organisational Change], which were the IMROC challenges at the time and we were adopting some of the challenges and bringing recovery focus care and recovery focus practice to the way, to the organisation of changes. And we were thinking well there you have a very set piece of coproduced, co-facilitated piece of work that’s, you know has been established so there has to be merit in it. CO4 (II) |
| B10 | I suppose there was a big push towards the recovery and really adopting it in to the service, especially when we moved in to our new building, and it just really fit in with everything that we were doing at the time […]. CO1 (II) |
| B11 | […] it’s really difficult to get staff in the acute services interested in what’s happening with EOLAS […] So there is a lot of disinterest I would say from our acute staff to engage with things that might actually prevent somebody coming back in and that’s a huge problem. CO3 (II) |
| B12 | I do think you know clinical leadership in a team is you know, is very important. Because it does set the tone. And I suppose it’s about buy in and attitudes and I suspect that you know every team is different. And you know I know that in our team like [name of psychiatrist] would be very enthusiastic about it. CF5 (FG) |
| B13 | Well we did get that buy in quite early on. But probably as well, for me because I suppose [Consultant Psychiatrist] was very supportive of it as well. That was our immediate link into the area management team from that level. Because I suppose I’m not of senior. CO7 (FG) |
| B14 | […] people were identified and they were told they were doing it, you know that kind of, I don’t mean told they were doing it, but we were all identified and then you know It was expected and you know you had to provide your figures and you had to do it and you had to get your cohort, you worked with your community nurse, you know there was a better level of commitment towards it. CO16 (II) |
| B15 | Depending on who is doing the, the consultant and writing and having the conversation with the person. And often, yeah and once they’re discharged it could be, no I’ve seen it, refer for EOLAS on discharge, refer to EOLAS, I have seen that on their discharge plans when I was working in the area. CO4 (II) |
| B16 | So coming back to the referrals, if you know, first of all you get your executive management team saying; yes we want this in our area. And this message gets out to the local sector teams. That they need to refer and this is the referral process. And this is the person that’s going to take the referrals. Then things happen at a reasonable pace. SH2 (II) |
| B17 | We just go into our managers. I don’t know who’s involved in our sector, we just went to our manager and said we’re going to be running an EOLAS in the evening, can we have the time back –  **Q: Okay, and managers went “yes”.**  Sure. CF9 (FG) |
| B18 | [...] but the biggest challenge we have from the EOLAS programme perspective, is in persuading services that this is something we really need to do. You know because you will find pockets of interest in lots of places and there’s some great people who really want to do it but you know they’re not supported then by the key decision makers within the service to prioritize this as a programme that can really make some difference. FF1 (II) |
| B19 | I have to say in terms of, although initially I’d say like buy-in from senior management was excellent it began to kind of decrease as we moved along yeah. […] The resources really weren’t there at that moment in time you know. So I kind of felt that definitely had an impact kind of mid flow of the programmes being rolled out but you know resources certainly weren’t as plentiful you know and management kind of saying it’s probably a luxury we can’t afford really at the moment you know. CF2 (II) |
| B20 | [..] even though there was only a certain few of us within the team going to do it. To be honest everybody kind of bought into it. You know because it did bring a sort of an energy to the place. And you know okay well what about this. So like our colleagues were very willing.  **Q: To get onboard as well and buy in?**  Yea which suggested you know, such a person might benefit from this. Or I know somebody, or what do you think. So it did bring a whole different energy within the place. CF12 (FG) |
| B21 | […] we had lots of support from medical colleagues. We had lots of support from our senior nurse managers and everyone kind of came forward around, you know that this programme [EOLAS] was a tripartite kind of approach to mental ill health. I suppose we would have had, we would have been lucky in the sense, I suppose we were such a new community service at the time that you know, everyone bought into EOLAS and what it could do. […] CF2 (II) |
| B22 | So some teams were slower in fairness [to refer to EOLAS], to you know buy in, or maybe I don’t know what it is. But I suppose maybe it’s to do just with the characteristics of the team. Or the maybe the consultant [...] CF5 (FG) |
| B23 | But there is other consultants unfortunately that avoid it and just you know, I would’ve been, like when I went out to sell it initially, I was kind of asked, well how much will I get paid to do this. That was the one question. I got another question, why does a service user have to know what they’re diagnoses is. CO11 (FG) |
| B24 | There was just one discipline involved, which was social work. […]. I think that one discipline is not sufficient. You need an active coordinating team to make things happen. SH2 (II) |
| B25 | […] you will find the dedicated few who actually buy into that. But when we’re, we’re speaking to the converted. We’re working with the converted all the time. So it’s to try and get to the people who have, for whatever reason, lost or are fatigued by all of this and just don’t believe that it can be done […] FF1 (II) |
| B26 | So there was great excitement and actually got off the ground very quickly, got buy in very quickly because it was the only thing. […] everybody was like let’s do this and let’s all get excited and the, the clinicians were excited as excited about it as the services and the carers so at the time it worked out brilliantly. CO13 (FG) |
| B27 | Again, I suppose to increase peoples’ buy-in because for like the majority of people you know we’re all so busy. You know there’s 10 different programmes running across the service and it’s hard to keep you know EOLAS to the forefront of peoples’ minds when you’ve got 4 different OT groups, WRAP [Wellness Recovery Action Plan] everything else happening as well. CF1 (II) |
| B28 | […] But the service user’s programme I would say, has competed more so with I suppose other programmes that are up and running in that sense that we’re drawing out of the same people all the time. CO7 (FG) |
| B29 | Yeah, a lot of our team, the rehab and recovery team, are trained, so it’s very easy to get swaps with people […] CF10 (FG) |
| B30 | We do have a couple of OTs [Occupational Therapists] that haven’t done full groups for me […] so I would use them to step in if, you know the facilitator needed a night off or couldn’t make it or whatever, step in. CO2 (II) |
| B31 | [names local service ] more than [names local service] was particularly good at the start. They had some very large groups. Very dependent on nursing and there were issues around release of staff. So that caused problems there. SH2 (II) |
| B32 | I suppose we had just the three [facilitators] and then one girl that was, she just wasn’t in a position to do it, and then, she had been doing it, it was just personal circumstances, and then I was on maternity leave, so we seem to have been down to one facilitator, then when I came back the other girl left and it was back down to one, so it’s just the, that seems to be the issue everywhere, talking to the other coordinators. CO1 (II) |
| B33 | Yea one of my facilitator was in the evening times, it was outside work hours. So in that sense it didn’t eat into clinical time, except you know preparation work might have a bit. […] It was just really managing your own workload really. That you balance it all out at the end of the day.  **Q: Well I suppose you all felt that you had the flexibility to manage your own workload?**  Yea we did. CF4 (FG) |
| B34 | I suppose we’re quite lucky in the independence that we’re given in our role to follow the initiatives that we feel is worthwhile to our service users really. So I suppose the only conversation I would have had with my direct line manager about it would have been, being released for the training days and obviously the subsequent time that it was going to take out of my clinical time rolling it out, facilitating groups and all that stuff. […]. I mean there was no issue around time allowed for training days, travel, even being approved to travel to training is you know increasingly becoming more difficult if it’s out of area. So yeah very supportive in terms of that. I suppose the logistical side of it. CF1 (II) |
| B35 | Well I think, again going back to time, like so we’re doing this as an add on to our clinical work and I suppose we’re exceptionally busy anyway, so finding the time to even, like we’re having a, a meeting today just, again just the, the three social workers, about this, that’s probably being cancelled three or four times because of our workload, you know, so we don’t have any protected time for EOLAS […] CO6 (II) |
| B36 | […] we still have our day hospital to run. And all our clients that are attending. So to do our working week and then find space to fit EOLAS in CF11 (FG) |
| B37 | […] we do use our own mental health, our own building, you know, in the evenings, […] And it works, yeah, it works quite well, we don’t have those kinds of issues in terms of safety and security CO15 (FG) |
| B38 | So there’s always battles about resources, finding even rooms and places to deliver EOLAS. There was hope that EOLAS would be delivered outside HSE premises. Because of the clinical connection, but no money, no nothing. SH6 (II) |
| B39 | There’s no app, or you know. System or excel or anything like that for maintaining a database. So we don’t have such. So that’s the thing. So that’s the practical thing. So if you ask the CNS [Clinical Nurse Specialist] who’s eligible to do EOLAS within your patient load. They won’t know. And how many of them have actually done EOLAS so far, well again they’ll be. They won’t necessarily be keeping track. SH1 (II) |
| B40 | […] really it needs a dedicated coordinator, it needs protected time, I think you could easy allocated half a day a week in terms of like maintaining a database, promoting it, seeking, sourcing referrals, the prep of the group, you know, all that administration, the, you know, the planning for it [...] CO6 (II) |
| **Domain C: Intervention Characteristics** | |
| C1 | I suppose prior to EOLAS we would have been running family education for years, in [names service ]. And both the co-ordinator [and I actually were involved in that. I suppose I felt personally that we needed something more, something with more guidance which was what the EOLAS has given us, in the structure to the programme. CO10 (FG) |
| C2 | […] it’s something we aspired to do but in terms of time commitments we weren’t able to kind of get that off the ground or weren’t sure what that would look like and how we could involve, particularly family and friends I suppose, but also service users, so it was a very structured way to do it. […] this was like a readymade package, if you like. Yeah. So it, it, that was definitely the most appealing part to it. CO6 (II) |
| C3 | […] somebody else has already kind of put together for us and kind of let’s go with that rather than kind of trying to reinvent the wheel. […] CO13 (FG) |
| C4 | [...] I think the book [the handbook ] was written too for people who don’t have a background [in clinical practice]. So the book I think was good. It wasn’t a difficult, well for me it wasn’t you know, it wasn’t difficult for me to understand what is in the book. SF11 (FG) |
| C5 | […] it’s [the manual] in layman’s terms I suppose, it’s all very black and white, you know that kind of way, it’s not kind of the doctors spiel on it, it’s written for people to be able to understand, yeah, it’s great, a bit of everything in it. FP7 (II) |
| C6 | […] the actual books [the manuals] are very good. They explain a lot and they’re you know well divided up and that. CF3 (FG) |
| C7 | Like there’s a lot packed into it [the manual] but it’s not too much and there’s a lot of information that is helpful. I found the booklet very good to have just to breeze over in my own time. SP (II) |
| C8 | **Q. Would you find it difficult doing it I suppose if there was no guidance there or no manuals [EOLAS training manuals]?**  A. I would find that difficult yeah because I would probably lose my track of, I find it hard to focus you know and keep to the point like. SF2 (II) |
| C9 | It [the manual] gives you guides you know, that you can let a room take off on its own you know. FF8 (FG) |
| C10 | And you can use kind of parts of it [the manual] to help get a conversation started. SF6 (II) |
| C11 | It [the manual] opens up the conversation in the EOLAS group. FP12 (II) |
| C12 | So we need to probably look at the referral process. I know it’s for a specific diagnosis and that’s very important and the programme is aimed for people like that. So you can’t just self-nominate or self-refer at the moment but maybe there could be some way of because I know people who would love to do the programme and have never even heard of the programme and I might just mention it and it’s complete news to them. FF1 (II) |
| C13 | And it just seems to be that EOLAS seem to have these constraints around it. Being referred only through the mental health teams, which is fine. Because you don’t want people coming incorrectly to the wrong course, or whatever. But I still don’t see why you can’t have posters up in GP [general practitioner] surgeries. Saying what the programme is, to contact the local coordinator […] FF10 (FG) |
| C14 | […] if we’re focusing on say recovery education, service user, family engagement to a specific area and if EOLAS isn’t fitting the criteria as in the referral, go back to the referral process. It will get lost in that. So the design is that we have one centre engineering and driving all education information for service users and family members, that it’s sort of a centre and it spreads out. So if EOLAS isn’t, if the referral thing is catch, if it doesn’t fit in there and it’s a standalone project, that it doesn’t come up for the recovery framework or our local education, recovery education plan […] CO4 (II) |
| C15 | […] it’s [EOLAS] not accessible through self-referral and that is extremely outdated, out moded and its anti-recovery in my view and it’s not recovery education at all, it is just not. CO5 (II) |
| C16 | […] if it’s [EOLAS] supposed to be recovery orientated and we’re trying to break the traditional way of doing things, it shouldn’t really be referral based. I know people, there’d be people who are discharged from the services who would love to do this programme you know and to give them a chance as well. SF4 (II) |
| C17 | And the, and one of the dilemmas about EOLAS being a referral only system and it being, and not being advertised because it is for a very particular group, is that people don’t hear about it in the way that they would hear about other programmes. SH5 (II) |
| C18 | […] if it [EOLAS] was more kind of advertised, advertised in your local shop, or in the local parish. Or that kind of thing that even your local GAA club that kind of, you know each side of the pitch. A lot more people in the community, to be able to come in and do a programme, like the EOLAS. SF6 (II) |
| C19 | And I think the key to it was that this [EOLAS] programme was evidence based. It had quite a bit of work already done and people were more enthused by that. […] I think they [executive management] realised that it had credibility as well. CO11 (FG) |
| C20 | Very much the fact that EOLAS was welcomed into service was the fact of the scientific research that was done on it. Because that impressed the clinicians. Because there was evidence of the benefits of the programme for family members, for service users and for the research also was very good on the impact on clinical people. And how they benefit from it. SH6 (II) |
| C21 | We arranged focus groups around [Irish county] for service users, for family members and service providers. Which was an opportunity for the project workers to just ask questions around like what do you think of this. What, how would you like it to run, what do you think would be most effective. What sort of information, learning material would be useful? You know how do you feel about you know facilitation and how do you think this facilitation should be done. How long should the meetings be for? What sort of venues would suit you best, all that sort of thing? So and likewise service providers you know, what do you think about education for service users. Would you be interested in facilitation? How do you think it should run all that sort of thing? So they did the initial scouting around of, to get the feedback from the ground. SH1 (II) |
| C22 | There’s a lot of other good programmes and they might be more specific to people. But EOLAS was only ever meant at the present time for people with psychosis. There is nothing else actually addressing people with psychosis and at that hopefully at that early stage. SH6 (II) |
| C23 | I think EOLAS would almost be the first stage before people start going in to recovery, I think they need that education piece before they start going in to other recovery programmes. CF9 (FG) |
| C24 | I would see it as a complementary thing to say, ARI [Advancing Recovery in Ireland] or WRAP [Wellness Recovery Action Plan]. And I think there’s no reason why somebody who’s done EOLAS cannot do ARI [Advancing Recovery in Ireland] and also do WRAP [Wellness Recovery Action Plan] also. SF1 (II) |
| C25 | […] you know when it’s so structured to that style, I find that can be counterproductive. Because something could come up that is week 3 or week 6 in week 2, but we’ll get to that but no they want the answers there and then. And that’s just from my own personal, I suppose delivering or facilitating groups, when I have set my aims, my agenda, my objectives for when people leave here they will have X, Y and Z, and that’s fine. That was where I was at until I sat down and stopped and listened to the groups of people that I was facilitating and going, the question is what is it you want to get from this group, what is it and you know whether its sessional pieces, whether it’s a full program or a stand-alone piece. I think we need to be more flexible in how we can deliver EOLAS. […] you know sometimes we’d be talking about how else can we deliver it here. […] CO4 (II) |
| C26 | And unfortunately I do have an issue then with the information as in it’s too static, the amount of work that goes into […] but that information is out date already. […] It’s a very one, you know it’s a one-dimensional information which again it’s just too static, it doesn’t fit everyone’s experience. […] CO5 (II) |
| C27 | Like each EOLAS event is slightly different due to the different participants. So there is a format there and there are topics that are covered but there is a lot of room to sway what exactly is discussed in each EOLAS session due to the people that are there. SP (II) |
| C28 | I think we did probably adapt it, like we found it really useful to have the booklets, to have a kind of preordained, as to this is what we’re going to do. But definitely, like the first couple of weeks anyway that I did with the families. We’d a lot of families who were really in the thick of it; you know their loved one was really unwell […]. We’d a lot of emotion; there was a lot of people just in tears you know. So you had to manage that and address it. But you couldn’t kind of by the way we have to; this is what’s in the booklet. So we now we still got through the stuff. But you know we just had to balance it, as to we had to deal with what was going on in the room. And then once the group kind of formed and settled, it was easier. CF11 (FG) |
| C29 | […]we felt it [the guest speaker psychiatrist session] was very medication focused. And if you have this, this is your diagnosis and this is the treatment. So at the end, we would always close off with these are other treatment options and these are things. So we kind of managed it that way. CF11 (FG) |
| C30 | I think it’s the idea of an eight-week programme. Yeah, it sounds very long and people are saying, oh, you know, on Monday I like to do this or I’m going to be missing a, you know, I’m going on holidays or... or there’s something coming up, I’ll miss a week […] CO1 (II) |
| C31 | I felt it was a long programme, it was long because it was at the time of day for me, you know, and it clashed with my work days, do you know, so it seemed like every week for eight weeks, it seemed like – and then there was, I think then there was Easter I think in between, so it was a bit of this is forever. FP9 (II) |
| C32 | It means for me as an ADON [Assistant Director of Nursing], like I hate when this group is on because I’m trying to, you know the way (laugh), there’s a huge amount of work, there’s a huge amount of work anyway because you’re trying to get the names, get them organised, organise the rooms, teas, coffees you know I do all that and I just really ask the facilitators to turn up and look after the clinical piece on the night. CO2 (II) |
| C33 | […] I coordinate it, and, like even though it doesn’t take a huge amount of time, but it takes a huge amount of time getting it up and running and I know we’re going to be talking about that, but the coordination of it, keeping the names and getting the speakers and that, but it’s not a huge amount, but at the same time if you’re very busy it is a lot, you know, when you break it down you sort of say Oh God, no, there’s not a lot, but when you try to keep it going it is. […] CO14 (FG) |
| C34 | […] the real challenge is the time pressures in terms of coordination pieces with their other role, that’s really the biggest problem for us […] it’s the stuff really around, you know, with our co facilitators maybe, you know, getting them set up, you know, in terms of getting our co facilitators set up, […]and making sure that they’re minded and looked after and that it’s a good experience for them has been hugely problematic. Some of the follow up stuff as well, just in terms of like I suppose the stats and research and stuff like that, like again that that creates, you know, […] it’s, it’s extra, it’s extra work after, like on top of, of the kind of core thing, which is you wanting to deliver that service, so, so that’s been, that’s been our kind of main issue. CO15 (FG) |
| C35 | I think the challenges for the time that it actually takes. And not that I, I have managed it like on top of a very busy workload. CO7 (FG) |
| **Domain D: Characteristics of Individuals (Provider Level)** | |
| D1 | You know there is a kind of a threat in the CPNs [Clinical Nurse Specialists], or that nurse feeling threatened by oh my god, I have to tell people about this. And if they ask me two questions about it I’m not going know the answer, I’ll look like an eejit. So maybe I just won’t bother mentioning it to them or something. CO8 (FG) |
| D2 | […] I suppose to tell a family or to advise a family that there is a group for. You have to know what the group is about. You have to kind of have done your own. And I suppose if the community nurse for example, was kind of, or wasn’t involved in the training. Wasn’t involved in direct kind of maybe didn’t, maybe there was a kind of a reluctance, or unconsciously. Not to maybe. I mean I suspect that if the facilitator was a community nurse. And they were out there, they would be selling it big time, you know. Whereas, I think and I don’t have any, I don’t blame the nurses, the community nurses. But when you’re hard pressed, to sit down with a family and to talk to them about the benefits. And the philosophy even and what kind of, what [is] this. CF5 (FG) |
| D3 | I just loved the whole concept of it and idea of it from the very beginning. So that’s kind of what, and I just felt there was such a gap there. CO14 (FG) |
| D4 | And I suppose my interest in EOLAS came about because there was nothing there. I could recognise that there was absolutely no support there for families. No information even certainly and as I say that’s from my experience you know. FF10 (FG) |
| D5 | Also, as the weeks went on also I’ve seen the value in it as well because especially having the lived experience there with the professional knowledge because you can see the lads, we do it actually in this room, and you can see the lads and they light up and they’re kind of saying, yeah that sounds very familiar that’s happens to me too. SF4 (II) |
| D6 | I would’ve done facilitation in a previous life kind of you know, in other roles and stuff like that. So I suppose I felt fairly confident in going forward for it. FF10 (FG) |
| D7 | […] I’ve worked in mental health for, God, I don’t know, eleven years or something, so, do you know, like for me, like I’m fairly confident in terms of that [facilitation] CO6 (II) |
| D8 | Were we ready? I suppose it’s only in the doing really that you really learn an awful lot. Very nervous the first few sessions, the first session in particular but in doing it the confidence rose and you know you become better at it. FF1 (II) |
| D9 | You see I worked as a HR person in the private sector. So I would have done facilitation skills and interviewing skills and coaching, you know and developing things. […]. So I would have had that experience and then yeah so and being involved in groups and running groups and I mean I would have done a bit of training in my role as well in my HR role. FF1 (II) |
| D10 | [...] so what they did in the facilitation [training] for the three days, it was like putting us into groups and you know then eventually towards the end, it was you yourself probably getting up and being able to facilitate with the little group that you’d been in or just directing the conversation and you know, or taking charge of maybe being the one that would get up and explain what you’ve discussed in the group. And that over the three days, gave you that encouragement. SF3 (II) |
| D11 | Then you’re sharing with others really what worked for you and you actually do create a very good rapport with people and it helps the group to participate then and that’s great. FF1 (II) |
| D12 | I think, again this particular lady was fantastic in terms of the group dynamics and I think was, it was, it came quite natural to us and we worked well together, but, I mean we had some people get upset and some people, and the co-facilitator was very able to manage that, or we were able to manage that […] CO6 (II) |
| D13 | I think it’s the content and you have to have a skilled and experienced facilitator that will be able to look at it and be able to probably amend it or adapt it to the group whilst getting the content delivered. I think that’s probably the key to it, knowing that to leave in or leave out or you know and trying to stay true to it as well I suppose is the thing, trying to stay true to the program you know. […] CO16 (II) |
| D14 | Well I’m definitely conscious of it that you know that there’s kind of the requirement for anybody who’s coming into an EOLAS group. To either give a talk or facilitate, is that you give up your power. You know you actually accept that these are human beings, you know, struggling with you know. And you have to be compassionate and you have to at least be able to empathise and relate to people where they’re at. CF5 (FG) |
| D15 | No. I tried very hard not to be the nurse. Not to be the manager within the facilitation. But I struggled in that. Because if someone didn’t show, if somebody wanted to stop their medication or whatever. My co-facilitator would be encouraging of that. And I found it very hard to stay in my role as a facilitator. And not as the nurse. CF12 (FG) |
| D16 | […] the service user facilitator has a much, it’s much harder for them to position than it is for us I think because there is, straddling that line between being a, being, receiving a service and facilitating within that service as well. And sometimes that, sometimes the difficulty of riding that line becomes really obvious, do I, am I here to tell my own story, am I here to, to be a, you know, here’s what you could be, kind of thing. And there’s one young woman who I’ve facilitated with a couple of times who, who finds that really, and she’s said it to me, she finds that really difficult. About where she, where she pitches herself I suppose, it’s, it is a really, really hard thing to do. CF7 (FG) |
| D17 | Well initially I would have kind of maybe taken a little bit of a back step. Because I would’ve considered that the, you know the, like the co facilitator being a health professional. Would have a certain set you know of knowledge obviously that I wouldn’t have. And I would’ve kind of you know, being, felt a little bit subordinate. FF9 (FG) |
| D18 | I suppose I’m self-employed, so I’m flexible. Otherwise I couldn’t do the middle of an afternoon, like you know and that’s the reality of it. FF10 (FG) |
| D19 | Yea well actually yea, well I’ve a good husband, if he wasn’t there I couldn’t do, go to these places you know like. Because my daughter can’t be left on her own, yea. FF4 (FG) |
| D20 | I had lined up for a course there in October, but then I got a job so I had to bow out you know. But I was kind of looking forward to doing it. And I thought at one stage I might be able to do it. But just the work schedule didn’t allow it. So I told [EOLAS Coordinator] that. SF10 (FG) |
| D21 | I’ve already lost a few service users […] lost from EOLAS […] I’d say for some of them, their own mental health probably deteriorated, one in particular mid one of the programmes and he hasn’t reengaged with EOLAS [….] A few family members have gone through very difficult times and pulled away from EOLAS. Not that EOLAS has been a trigger for anything. But just their lives have been difficult at different periods of time, so. […] CO7 (FG) |
| D22 | […] the other social worker trained, again was really motivated and found referrals herself.. CO5 (II) |
| D23 | And the success, I have to put down to the work from the facilitators around the table you know the key people, the contact people just keeping it on the agendas. CO4 (II) |
| D24 | I’ve had some very, very good and strong family members who came onboard from the very beginning and they’re still onboard. […]. I have been very lucky with particular family members. I have that one or two of them could’ve been the coordinator of EOLAS themselves. You know it’s just you know they really have always been there and always ready to deliver and you know. CO7 (FG) |
| D25 | There’s only 1 or 2 [MDT members], it’s like what I said about the service users, there’s only 1 or 2 I suppose who would be dedicated and would be committed. Yeah, so everybody else kind of, you wouldn’t get much of a response you know. CO16 (II) |
| D26 | […] there's a core of us who are very committed, you know, and have a passion for it but most other people just it's a job, they’ve got families, they have, we have to recognize that. SH4 (II) |
| **Domain E: Implementation Process** | |
| E1 | Yeah, they’re [EOLAS], like they are very good, your, you know, they’re very accessible, if there’s every a question, you know, you can get through to [EOLAS project worker 1], [EOLAS project worker 2]or [EOLAS project worker 3] previously, when he was there, they were very good, very accessible […] CO1 (II) |
| E2 | […] we berate ourselves all the time but support from the national, you know from [EOLAS Project Worker 1] and [EOLAS Project Worker 2] in the past and [EOLAS Project Worker 2] and [EOLAS Project Worker 3] is great, I mean they’re always contactable or always there you know really are, they’re great. CO4 (II) |
| E3 | And we had meetings outside the EOLAS to plan when we’re going to have the next one. And we were all invited and organised who would be available to do it. FF8 (FG) |
| E4 | […] we set up a steering group. With [CO8 (FG) – Consultant Psychiatrist] was on it, and there was team coordinators from each area. Because what have we five, five areas, five teams within our area. So we had kind of the key people, in terms of. Yea from each area and straight off with myself and then with one or two of the clinicians. And one or two of the family members and the service users, were part of that steering group as well. CO7 (FG) |
| E5 | And I would have initially set up a meeting I suppose with the people who were trained, the lived experience people, the family carers and the service providers and to see what had been done to date I suppose, just a planning meeting. And to see where could we go and where were we going to take it, what were our plans for this few months going forward, how were we going to capture our cohort. So a usual, I suppose a normal kind of a planning meeting. CO16 (II) |
| E6 | So I think the, in terms of the coordinators, they have to have that seniority, they have to have the time and the commitment to do it and the actual dedication and the belief in EOLAS to do it. The belief in the value of it. They also need to be really good organizers and persuaders because a big part of this role is persuading people to do things for you, whether it's either making a referral or even just teams having EOLAS on their agenda or its facilitators being available or sorting out the [payment] register, there's a lot of negotiation and moving that actually needs to happen. SH05 (II) |
| E7 | So I think what we realised is that whoever that coordinator is going to be, needs to have some status. Whether it’s a senior social worker, assistant director of nursing, senior OT whatever it is. But they need to be influential […] they need to have that ability and that experience of managing people and if you don’t have that experience of managing people and managing resistance. Then you’re going to find it really, really difficult. And that was the case, basically everywhere you know. SH02 (II) |
| E8 | I organise all the bits, all the pieces, I organise the docs, the manual, all that sort of stuff. Because I think people are really, really busy, the clinician are really, really busy and its enough for them to get to the group and deliver the group and do all the bits with their other facilitators. […]. It means for me as an ADON [Assistant Director of Nursing], like I hate when this group is on because I’m trying to, you know the way (laugh), there’s a huge amount of work, there’s a huge amount of work anyway because you’re trying to get the names, get them organised, organise the rooms, teas, coffees you know I do all that and I just really ask the facilitators to turn up and look after the clinical piece on the night. CO2 (II) |
| E9 | The coordinator does most of it [recruitment of attendees] like, within our team we would ask people ourselves who we were working with, but with regards to the, I’m on a rehab and recovery team, so the community teams, the coordinator would be contacting those teams. But I found that really helpful and I think there’s a lot less pressure when it’s just turning up basically.. CF10 (FG) |
| E10 | Yeah so I suppose I took on the role of getting the referrals you know talking to the team, getting buy-in from the team, finding a venue, that kind of logistical side. I did that for the area. I suppose the EOLAS coordinator took on the challenge of getting a service user and family member from [Large town in ES07] signed up to travel to [2nd town in ES07]. CF1 (II) |
| E11 | We’d probably have a bit of both, the coordinator would send out the kind of overall email to the whole service and would organise the guest speakers, would prompt, kind of this is the date, and then it’s the ground work then of kind of collating all the referrals and the organising the stationary and that kind of stuff. So it’s a bit of both. CF6 (FG) |
| E12 | It seems to be psychiatry, that’s where the power is so we’ve got to get psychiatrists on board with it and how do you do that? FF1 (II) |
| E13 | I just remember one conversation with a, a consultant and she told me that [EOLAS steering committee member – Consultant Psychiatrist] had come in and spoken to the consultant group and it was like a light bulb moment for her, even though I had spoken to the group as well, but, I don’t know whether I was talking French or German or something but […] I think by [EOLAS steering committee member – Consultant Psychiatrist] coming in it gave it the stamp of approval. CO14 (FG) |
| E14 | […] what we did after our first EOLAS experience, we asked, you know the way the trainee doctors and the consultants have their forum every week where they have to present something, we asked could we come in with the family members and the service users and give a presentation on the experience - to kind of sell it to them for the coming years, and that seemed to work a treat […] CF9 (FG) |
| E15 | […] I did up a summary of activity and, so he’s [consultant] going to bring it to the consultants’ meeting and I asked him just to look over it and see what he thought of it and he gave me suggestions as to what to change, because they all love stats, and it’s really quantitative, what happened, and what happened to the people, you know, that you sent out the letters that you didn’t hear anything from, you know, and what was the result, so I did that, but I haven’t had anything back as yet. […] I included also, I mean some lovely qualitative stuff that we’d got from the service user experience […] CO12 (FG) |
| E16 | The consultant wasn’t leading it, or were, they were giving it the nod. But not actually proactively giving it the nod. Because I had to go back to those areas and say, listen if you don’t put in the referrals, we are going to scrap this. CO11 (FG) |
| E17 | I think the psychiatrists haven’t just got it yet if that makes sense. I just think but then you see when you go in to see a psychiatrist they have a set amount of questions right and they might come to the 6th one or whatever and somebody might have said to him maybe somebody might need EOLAS but I’d say that’s way down their list of priorities. SF8 (II) |
| E18 | […] it was all nursing actually who were delivering and co facilitating the programmes. And they were able to recruit facilitators because they had engaged in the community. They knew family members and service users to come, to the facilitation training. SH06 (II) |
| E19 | I was on a community team at the time so it was to get service users and family members from my director who I knew and I knew might either benefit from EOLAS or be interested in it or who would have expressed an interest in you know giving back or doing something more because they had gotten, you know whatever the reason was they had experience or they wanted to do something. CO5 (II) |
| E20 | […] the particular individual who actually came with the invitation or asked me to get involved, she’s a service provider and I would have a lot of faith in her and she described it really well. So that would be number 1 why I said, right I trust this individual and if there’s anything she particular promotes well then it’s worthwhile. FF1 (II) |
| E21 | I would’ve done some work with the nurses and stuff that were involved with ARI [Advancing Recovery in Ireland]. So it was them that kept kind of pushing me to do it. Because obviously they saw, kind of something in me. But it was great to have I suppose that bit of a push SF6 (II) |
| E22 | We do meet every two weeks and we would go through say the number one, number two. And we’d talk about right, who’s going to do that who’s going to do that role. FF11 (FG) |
| E23 | So for the first session that we did, we met up probably half an hour before we went into the session. And [Name of cofacilitator] would say, well I’ll take this would you like to take that. And we shared taking each part. And then what we would do after every session after that we would stay behind probably for about fifteen, twenty minutes. And we’d go through the same pattern. And we’d say and then we’d go away and we’d take that part of it. And you know we’d structure it ourselves. So that it was ready for the next week. SF3 (II) |
| E24 | And then I suppose the planning meetings then as a whole group, meeting beforehand, before the groups were even ran at all, making sure that we all met beforehand and that everybody was comfortable with it. And that people were happy to work together and all that kind of stuff. You know and then just being really clear to try and keep everything as balanced as possible, so encouraging the facilitators and especially the clinicians to be open and to divide the work and to kind of, you know offer to take the bits that maybe are more difficult, like literally access to buildings, you know sending out you know invitation letters that obviously the clinician should do that because they can get the postage paid for and all the rest. But making sure that you know the letter is actually drafted by both facilitators so making sure it is as equal as possible and coproduced as possible. CO5 (II) |
| E25 | […] I checked in enough that if somebody wasn’t doing well because like do you know after maybe the first few years there was a point where, you know 1 or 2 people either wanted to step back as in the peers or became unwell or whatever it was, that I checked in enough that I caught those things […] CO5 (II) |
| E26 | I suppose the service user that I am co-facilitating with […] I suppose he was experiencing quite a bit of anxiety about taking the lead on certain activities and that. […]. So yeah like even before groups when we would meet and you know discuss the content and all of that. I suppose because of his anxiety I felt a little bit more, well you don’t worry about that I’ll look after that. So you know I feel like maybe I’ve been doing more than a 50/50 split in terms of the workload I suppose. CF1 (II) |
| E27 | […] there’s a bit of politics involved in some sense because people are eager to do it, you know, people are interested in it and also then there is the payment with it, so we are very conscious that you don’t, you know, that, I suppose it’s fairly done......that it’s done with, you know, and especially when training or anything comes up and, you know, you are always conscious that this person has done it before and maybe this person would like to do it and, you know, so there’s, there’s that type of just basic, they’re kind of like nearly housekeeping type things CO1 (II) |
| E28 | I think there’s a good awareness [of EOLAS] in [urban town] because [clinical facilitator] champions it so well. I know there’s a few other people that really champion it really, really well [Assistant Director of Nursing] and a few people like that. SF8 (II) |
| E29 | Having staff in the area is very important, like I have two social workers and a nurse, or two nurses in that central sector. And then in that other sector I have actually two nurses, two community mental health nurses. One CMN2 sorry and a community mental health nurse. And because they are at the meetings, they’re kind of bringing it up regularly. The conversation gets had. Whereas, in the other places it’s not there. So that not having the ongoing conversation around it and saying, well listen have we any referrals or whatever. And it keeps the focus of attention. CO11 (FG) |
| E30 | **Or have you seen sites that you think actually what they have done has been really clever, what they’ve done has been an integral part of how they’ve got this up and running?**  Number one – there’s a champion. All right. And that there are probably multiple champions in that you’ve a coordinator who’s a champion and is really taking a management role in the delivery of it so they’re actually seeing it as a project to be delivered in its complexity. And that where it really works is then where that person has the support of the other clinicians so that there’s buy-in from the teams in terms of that this is a resource that they’re using. SH05 (II) |
| E31 | It is champion lead. And that is a sustainability risk, you need to build the teams around you. It’s not, you need to have people who know about EOLAS, who are willing to do EOLAS. To refer, send people on to you automatically. SH06 (II) |
| E32 | For EOLAS to sustain itself there has to be champions in each area. So there has to be some sort of succession planning. And I suppose, I can give you an example, in we’ll say one particular area where a director of nursing was the champion for EOLAS. And retired and now there’s no champion in the area. SH02 (II) |
| E33 | I suppose people are constantly moving as well in terms of staff. You know there’s people moving into different roles, there’s people moving out of area into different roles. So I mean if I was to take a job somewhere else next year, as it stands if we didn’t have someone else on the team trained it probably wouldn’t happen. So I suppose it’s kind of planning for that as well. CF1 (II) |
| E34 | […] when I circulated it [evaluation] everybody on the teams knew who referred in to EOLAS. Who was the person that actually did it? So you were kind of saying, well listen you know You’re getting credibility for doing that (Laughs) and that was a good idea. Because right away and I circulated it widely kind of I just let it go. And plus the fact the feedback on, it was qualitative and quantitative right. So right away people, even the consultants themselves were getting an understanding and direct feedback. As to what the families were saying and what service users were saying. Which in turn, actually, increases the potential possibility of them referring in, having more engagement with it and seeing the benefit of it. CO11 (FG) |
| E35 | But I know after our first program [name of coordinator ES08] done an evaluation actually in [ES08] and presented the feedback to the management team. And you know broke it down and just people’s comments and you know it was done anonymous and just our numbers and things like that and sent that down through the system. And it definitely generated more referrals for like you know, we ran a spring program and now an autumn program and it definitely generated more numbers and more information and you know because there’s lots of programs happening. SF14 (FG) |
| E36 | I’m one of these people who likes to prepare and it’s important to prepare and then, so you do learn from one session to the next or what happened. So you might have a little bit of feedback and it’s great to be honest with each other. If you can do that to be honest with each other you know; what did I do that I shouldn’t have done or is there something that I could have done to make it easier for you? FF10 (FG) |
| E37 | And then there was the evaluation. Which is you know, just getting surveys, exactly feedback and focus groups again, just to get feedback. And then we, so that was the first kind of year really. So then it was a matter of sitting down with the feedback and rewriting the whole thing again for publication. The actual handbooks weren’t prepared until after that initial round. So that was another, I won’t tell you, I won’t tell you how much time it took to do it. SH01 (II) |
| E38 | Do you know that who knew the handbooks were going to take so much longer? And we didn’t know and we couldn’t know that until we started it. And low and behold it took longer than planned. And that completely compromised the possibility of proceeding with the train the trainers as planned. And there was no way around that that’s just the way it was. So that task had to be deferred. SH01 (II) |

Codes: CO= Coordinator; CF= Clinical Facilitator; FF= Family Member Facilitator; SF= Service User Facilitator; FP= Family Member Participant; SP= Service User Participant; SH= Stakeholder; II= Individual Interview; FG= Focus Group Interview.
